# Hitting the right note at the right time: Circadian control of audibility in *Anopheles* mosquito mating swarms is mediated by flight tones

**DOI:** 10.1126/sciadv.abl4844

**Published:** 2022-01-12

**Authors:** Jason Somers, Marcos Georgiades, Matthew P. Su, Judit Bagi, Marta Andrés, Alexandros Alampounti, Gordon Mills, Watson Ntabaliba, Sarah J. Moore, Roberta Spaccapelo, Joerg T. Albert

**Affiliations:** 1 Ear Institute, University College London, 332 Grays Inn Road, London WC1X 8EE, UK.; 2 Francis Crick Institute, 1 Midland Road, London NW1 1AT, UK.; 3 Division of Biological Science, Nagoya University, Nagoya, Aichi 464-8602, Japan.; 4 Institute for Advanced Research, Nagoya University, Nagoya, Aichi 464-8601, Japan.; 5 Vector Control Product Testing Unit, Ifakara Health Institute, Ifakara, Tanzania.; 6 Epidemiology and Public Health Department, Swiss Tropical and Public Health Institute, Socinstrasse 57, Basel 4051, Switzerland.; 7 Department of Medicine and Surgery, Centro Universitario di Ricerca sulla Genomica Funzionale (C.U.R.Ge.F), University of Perugia, Perugia, Italy.; 8 Consorzio Interuniversitario Biotecnologie (CIB) Trieste, Italy.

## Abstract

Mating swarms of malaria mosquitoes form every day at sunset throughout the tropical world. They typically last less than 30 minutes. Activity must thus be highly synchronized between the sexes. Moreover, males must identify the few sporadically entering females by detecting the females’ faint flight tones. We show that the *Anopheles* circadian clock not only ensures a tight synchrony of male and female activity but also helps sharpen the males’ acoustic detection system: By raising their flight tones to 1.5 times the female flight tone, males enhance the audibility of females, specifically at swarm time. Previously reported “harmonic convergence” events are only a random by-product of the mosquitoes’ flight tone variance and not a signature of acoustic interaction between males and females. The flight tones of individual mosquitoes occupy narrow, partly non-overlapping frequency ranges, suggesting that the audibility of individual females varies across males.

## Teaser

By beating their wings faster around sunset, male *Anopheles* mosquitoes sensitize their auditory system to female flight tones.

## INTRODUCTION

In many sexually reproducing animals, two mating types, or sexes (most commonly, males and females), must find each other for the act of copulation. However, both extrinsic (e.g., environmental) and intrinsic (e.g., sexual dimorphism–related) factors can lead to asymmetries in the spatial and temporal dispersal of the sexes (*1*). This is also true for disease-transmitting mosquitoes (*2*). Mosquitoes display numerous sexually dimorphic traits, including the female-specific blood-feeding behavior (*3*) and the male-specific daily formation of mating swarms (*4*). Mating swarms, however, are also part of the solution to the dispersal problem: hundreds or thousands (*5*) of males congregate at a fixed location, guided by visual markers (*6*), and at a fixed daytime, typically dusk (*7*), to act as reproductive mates for a much smaller number (a few dozens) of sporadically entering females.

In the malaria vector species of the *Anopheles gambiae* complex, mating swarms form a crucial reproductive bottleneck, making them a prime target of current vector control efforts (*7*). While *Anopheles* mating swarms can form reliably at the same sites, and same daytimes, for years on end, individual swarms have durations of sometimes less than 20 min (*8*, *9*), which also makes them an astonishingly ephemeral phenomenon. The short-lived nature of the swarms together with the sparsity of females is intriguing from two scientific points of view. Chronobiologically, it implies a tight synchronization between male and female activities. Neurobiologically, it suggests a highly efficient operation of the sensory systems that guide the males’ mating behavior. A key sensory modality for a male’s copulatory success is his sense of hearing (*10*). In mosquitoes, copulae between male and females form in midair and are preceded by an acoustic chase, where a male follows the flight tone of a female. This long-known male behavior (*11*), called phonotaxis, must succeed against the backdrop of the flight tones of hundreds of other males and constitutes one of the most reproducible, and most impressive, behaviors in insects.

Mosquito hearing relies on an active process (*12*); the flagellar sound receivers pick up airborne vibrations (*13*–*15*), which are transduced into electrical currents, and mechanically amplified, by mechanosensory neurons of Johnston’s organ (JO) (*11*). Together, JO and flagellum form the mosquito flagellar ear (for short, mosquito ear). The JO of an *A. gambiae* male is exquisitely sensitive and responds to flagellar tip deflections of <20 nm (or <1 mdeg, respectively) (*16*). For a mosquito, however, hearing goes beyond the simple reception of external sounds, as it also involves, and partly necessitates, the generation of sound. The mechanistic explanation for these settings lies in the way mosquito hearing works (*17*). The operation of the mosquito ear introduces essential nonlinearities [e.g., gating compliances (*16*)] into the mechanics of its flagellar sound receiver. As a result of its nonlinearities, a stimulation with two pure tones will generate additional, mathematically predictable distortion products (*18*) in the receiver’s motion. For the ear of a flying male, some of the lower-frequency distortion products that are generated by the mixing of his own flight tone with the flight tone of a nearby flying female will be more audible than the actual flight tones themselves (which are mostly inaudible) (*19*). Hearing, or more broadly audibility, in mosquitoes is thus inextricably linked to their flight activity and dependent on a specific interrelation between male and female flight tones and the distortions that these produce (*20*).

One such relational state, described as harmonic convergence (*21*), is the transitory matching of male and female flight tones at the level of higher harmonics. Mosquito wingbeats are not “pure,” and their flight tones comprise a set of sinusoidal waveforms, which oscillate at distinct frequencies: one at the fundamental frequency, equal to the wingbeat frequency, and several at higher harmonics, which are frequencies equal to integer multiples of the fundamental. Harmonic convergence has been interpreted as acoustic interaction between males and females (*22*). To investigate the relationships between harmonic convergence, distortion products, and the acoustic environment within swarms, we surveyed the daily flight tone landscape of *A. gambiae*, specifically probing for circadian modulations of audibility related to the mating swarm (*23*). We also quantified the relative audibility contributions made by males and females, respectively.

Our data show that males, but not females, increase their flight tone frequencies at the time of swarming, i.e., around sunset. The male-specific frequency increase is at least partly driven by the circadian clock and independent of any interaction with a female mating partner. It is also seen in males kept individually. The swarm-related modulation lifts the males’ flight tone frequency to 1.5 times the mean female frequency, thus exploiting an optimality of the mosquitoes’ distortion-based hearing system and maximizing female audibility during swarming. The fact that the mosquitoes’ flight tone–mediated audibility control is centered on a male/female ratio of 1.5 also explains the previously reported occurrence of “harmonic convergence” events, i.e., the short-lived numerical match of the second harmonic of the male flight tone with the third harmonic of the female flight tone. Harmonic convergence was interpreted as a signature of acoustic interaction, or communication, between male and female mosquitoes. At a fundamental flight tone ratio of 1.5, though, harmonic convergence occurs by default, independent of any interaction. We also conducted an in-depth statistical analysis of an existing harmonic convergence database. All occurring harmonic convergence events were indeed fully explained by chance and did not provide evidence for acoustic interaction between the sexes.

## RESULTS

### Tight circadian synchrony between the sexes

To probe behavioral synchronicity between the sexes, we monitored baseline activities (1 min; binning; LAM25H-3, TriKinetics) separately in *A. gambiae* males and females. Both sexes were exposed to the same environmental sequence: Three initial days of light:dark (LD) entrainment [consisting of 11-hour-long days and nights, flanked by 1-hour-long artificial “sunrises” and “sunsets,” at 28°C and 80% relative humidity (RH)] were followed by 5 days without any temporal cues (“free-running conditions”: complete darkness, at 28°C and 80% RH).

**Fig. 1.**
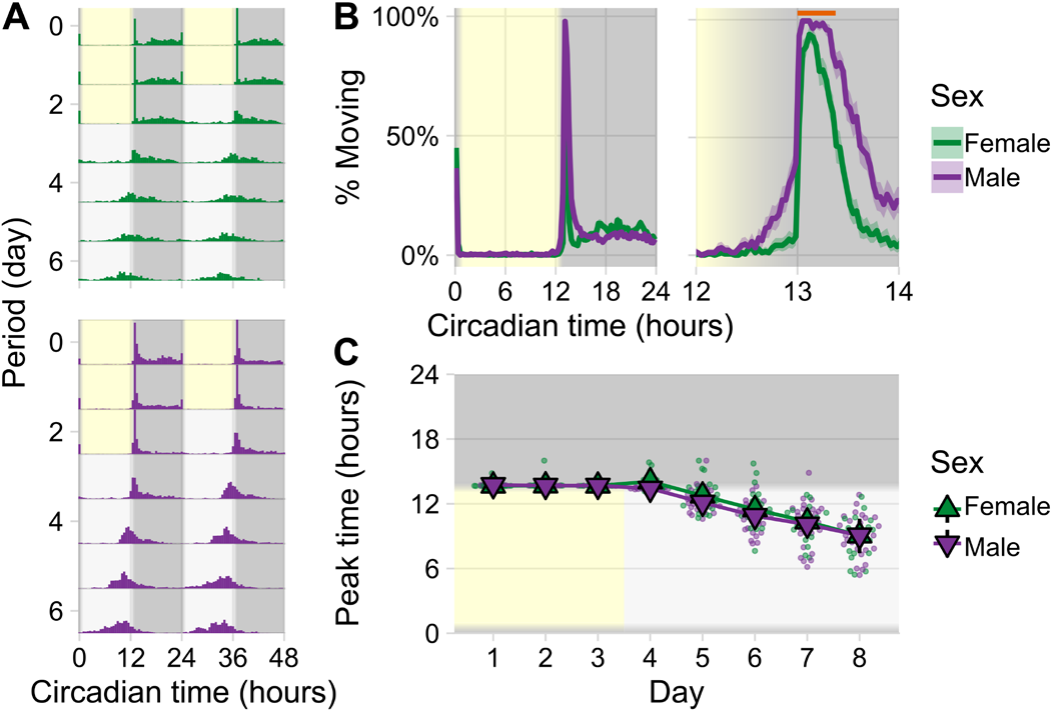
Circadian locomotor analysis of mosquitoes held in activity monitors (TriKinetics) housed in single-sex groups of three in glass activity tubes. (**A**) Double-plotted actograms for entire experiment: three days of 12-hour light/12-hour dark entrainment (with 1 hour dusk/dawn simulations) indicated by the yellow (day) and dark gray (night) background at 28°C followed by 5 days of free-running conditions, where the light gray background indicates the previously entrained day phase of the experiment and the dark gray background indicates the previously entrained night phase, i.e., constant darkness and constant temperature. Activity data are binned at 30 min and plotted in overlapping 48-hour intervals to visualize periodicity. (**B**) Average locomotor activity across three entrainment days (left: for entire 24 hours; right: for 2 hours around dusk). Activity is plotted as percentage of groups moving ±SEM. Orange bar indicates period of high activity. (**C**) Daily peak activity times across the entire experiment, calculated as time of highest activity from low-pass–filtered raw data (>30 min). Triangles display group means ± 95% confidence interval (CI) (*n* = 20 female tubes and 27 male tubes). Data are shown from one experiment.

Under light:dark conditions (i.e., with light changes acting as the “time giver” or Zeitgeber), the activity patterns of males and females were highly similar (Fig. 1): An initial “lights-on” startle response at sunrise was followed by a near complete inactivity during the rest of the day (Fig. 1A). Main activities were shown at sunset. Males started their activity increases earlier, and maintained them for longer, than females (Fig. 1B). Both sexes responded to lights off (at Zeitgeber time 13 hours, or ZT13, and coinciding with an illuminance drop from ~20 lux to zero) with an immediate and steep activity increase. The males’ activity plateau lasted for <30 min and fully enveloped the female activity peak (Fig. 1B). During entrainment, the males’ and the females’ peak activities differed by ~1 min (Fig. 1C). Even in the absence of external temporal cues, male and female activities remained tightly synchronized. On the fifth day of free-running conditions, the time difference between male and female peak activities was only 16.93 ± 35.12 min SEM (*P* = 0.63, Welch two-sample *t* test; female tubes = 20, male tubes = 26), and the 5-day average difference between the sexes was less than 30 min (23.00 ± 12.47 min SEM; *P* = 0.14, Welch two-sample *t* test; *n* = 5 days) (Fig. 1C). The synchrony between the sexes persisted, although their circadian clocks ran considerably faster than 24 hours (free-running period in males: 22.64 ± 0.12 hours SEM; females: 22.54 ± 0.11 hours SEM; fig. S1), and thus, activity peaks themselves constantly shifted to earlier daytimes (Fig. 1C, bottom).

### Male-driven audibility boost in swarming *Anopheles*

**Fig. 2.**
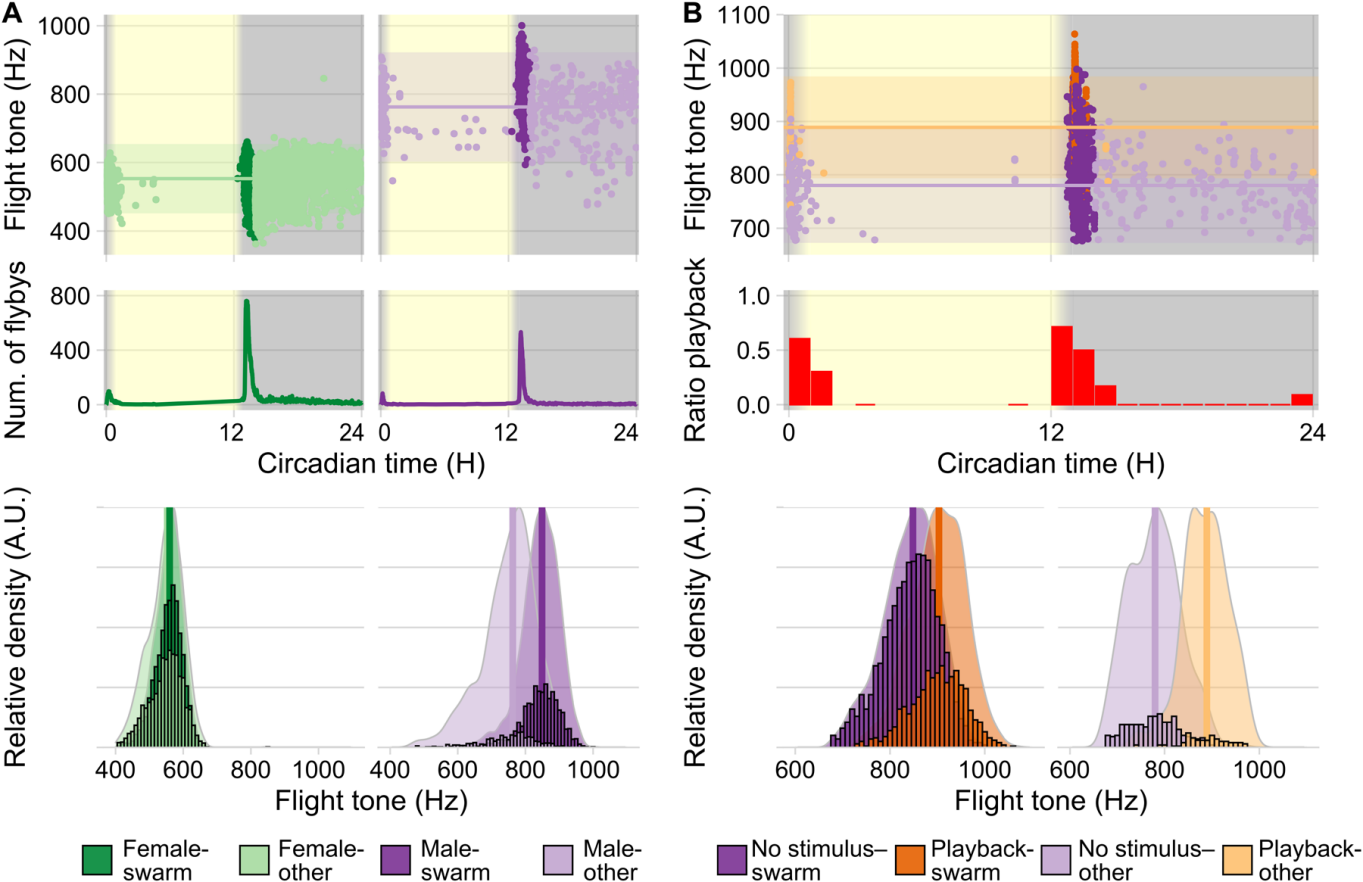
Acousto-circadian analysis of free-flying *Anopheles* (~100 single-sex individuals in 30 cm–by–30 cm–by–30 cm “bug dorms”). (**A**) Top: Female (green) and male (purple) flight tones during 12-hour light/12-hour dark entrainment (1-hour dusk/dawn) at 28°C. Points represent median flight tone frequencies of individual flybys; darker colors mark swarm time. Horizontal lines show population means for out-of-swarm (other) flight tones ±95% CI (female = 547 Hz, σ = 51 Hz, *n* = 2339 flight tones; male = 751 Hz, σ = 80 Hz, *n* = 445 flight tones). Middle: Running averages (5-min window) of flyby events as activity measure. Mean peak activity times across all days: female = 13.18 H, σ = 0.01 H, *n* = 12 days; male = 13.15 H, σ = 0.01 H, *n* = 18 days. Bottom: Bar plots of individual (male/female) flight tones plotted against scaled density plots show distribution shifts between the phases: swarm and out-of-swarm (other). Vertical lines show respective means (female-swarm = 556 Hz, σ = 40 Hz, *n* = 3040 flight tones; female-other = 547 Hz, σ = 51 Hz, *n* = 2339 flight tones; male-swarm = 844 Hz, σ = 55 Hz, *n* = 1600 flight tones; male-other = 751 Hz, σ = 80 Hz, *n* = 445 flight tones). (**B**) Top: Flight tones of males presented with a 1-min artificial female flight tone (550 Hz) at 30-min intervals. Middle: Flyby events during playback as a proportion of total number of flybys within each interval. Bottom: Bar plots of individual flight tones plotted against scaled density plots show distribution shift between playback and no-playback groups for each phase. Vertical lines show respective means (no stimulus–swarm = 843 Hz, σ = 58 Hz, *n* = 2318 flight tones; playback-swarm = 900 Hz, σ = 58 Hz, *n* = 928 flight tones; no stimulus–other = 779 Hz, σ = 54 Hz, *n* = 354 flight tones; playback-other = 886 Hz, σ = 48 Hz, *n* = 92 flight tones). Data pooled across independent experiments (females = 3; males = 4; playback = 4). A.U., arbitrary units.

A very close temporal alignment was also seen in the activity peaks of free-flying populations of *Anopheles* males and females (single-sex cages of 100 mosquitoes). When assessed in separate cages, male and female activity peaks (quantified acoustically by the number of flyby events past a stationary microphone; Fig. 2, A and B) differed by only 1.50 ± 0.25 min SEM in light:dark and by 1.75 ± 3.50 min SEM on the first day of free-running conditions (fig. S2).

Male, but not female, flight tones varied greatly across the day (Fig. 2, A and B). At 28°C, under light:dark conditions, male flight tones had a mean frequency of 844 Hz (σ = 55 Hz, *n* = 1600 recorded flight tones) during swarm time (±30 min around the circadian sunset at ZT13) compared to 751 Hz (σ = 80 Hz, *n* = 445 recorded flight tones) during all other times of the day (Fig. 2A, right). The corresponding increase of 93 Hz between the means is both biologically and statistically significant (*P* = 1.50 × 10^−84^, Welch two-sample *t* test; Cohen’s *d* = 1.53). Female flight tones, however, only showed negligible daily variations (Fig. 2A, left). During swarm time, the females’ mean flight tone was 556 Hz (σ = 40 Hz, *n* = 3040 recorded flight tones) and 547 Hz (σ = 51 Hz, *n* = 2339 recorded flight tones) during all other times of the day. While this difference is a statistically significant increase of 7 Hz between the means, the respective effect size, and corresponding biological significance, is negligible (*P* = 1.03 × 10^−10^, Cohen’s *d* = 0.18). The ratio between male (*f*_2_) and female (*f*_1_) flight tones was 1.38 ± 0.20 for most of the day, but the male-specific increase lifted it to a value of 1.53 ± 0.16 around sunset.

In line with their daily flight activity patterns, populations of male *Anopheles* showed phonotactic responses (measured as increases of flyby events past the source of a 550-Hz pure tone) predominantly at swarm time, i.e., at sunset (Fig. 2B, top). Some, albeit reduced, phonotactic responsiveness was also seen in the morning (at sunrise), but responses were virtually absent throughout the rest of the day (Fig. 2B, middle).

After playback of a female-like tone (550 Hz), the mean flight tones of male *Anopheles* rose further to 900 Hz (σ = 58 Hz, *n* = 928 recorded flight tones) during swarm time (Fig. 2B, bottom left). This phonoacoustic response lifted the male/female flight tone ratio to 1.62 ± 0.2. The flyby increases shown by *Anopheles* males after playback at other times of the day also associated with an upshift of their own flight tone frequencies (Fig. 2B, bottom right), suggesting a close link between phonotactic and phonoacoustic response. The key behavioral changes observed around ZT13, i.e., the increases in flight activity and flight tones, persisted under free-running conditions, demonstrating their circadian origin (fig. S3).

**Fig. 3.**
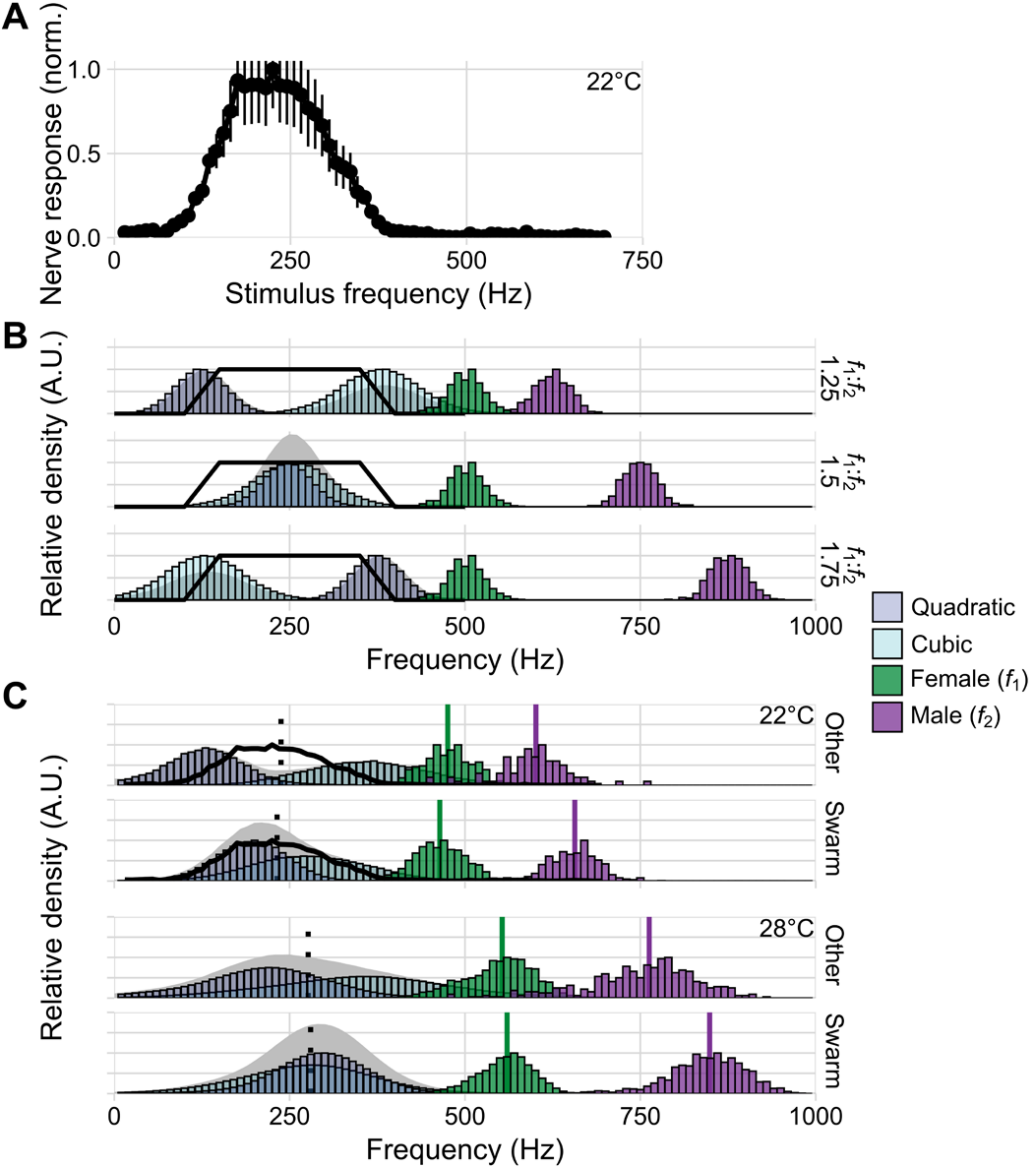
Flight tone–dependent generation of audible distortion in male *A. gambiae*. (**A**) Responses to pure tones (15 to 695 Hz, 10-Hz resolution) from antennal nerves of males (medians ± SEM; *n* = 7; temperature, 22°C). (**B**) Conceptual plot illustrating distortions (quadratic, *f*_2_ − *f*_1_; cubic, 2*f_1_ − *f*_2_) produced by mixing the flight tones of one hypothetical female (*f*_1_; μ = 500 Hz, σ = 25 Hz, *n* = 500) with three alternative males (*f*_2_; μ = 625; 750; 875 Hz, σ = 25 Hz, *n* = 500). Solid black lines show hypothetical nerve sensitivity range. At *f*_2_/*f*_1_ ratios of 1.5 quadratic and cubic distortion tones superimpose at a frequency of 0.5*f_1_; note the audibility effects of the superposition. (**C**) Distortions calculated from our experimental data for swarm and out-of-swarm (other) distributions. For 22°C, our nerve data (solid black line) can be compared to the created distortions (dotted line: predicted optimal frequency of 0.5*f_1_). At both 22° and 28°C, the overlap between the two distortion tones is greater at swarm time. Colored vertical lines show corresponding mean flight tones (22°C-other: females = 475 Hz, σ = 37 Hz, *n* = 447 flight tones; males = 595 Hz, σ = 52 Hz, *n* = 83 flight tones; 22°C-swarm: females = 464 Hz, σ = 37 Hz, *n* = 1137 flight tones; males = 653 Hz, σ = 39 Hz, *n* = 454 flight tones; 28°C-other: females = 547 Hz, σ = 51 Hz, *n* = 2339 flight tones; male-other = 751 Hz, σ = 80 Hz, *n* = 445 flight tones; females = 556 Hz, σ = 40 Hz, *n* = 3040 flight tones; males = 844 Hz, σ = 55 Hz, *n* = 1600 flight tones). Average male/female flight tone ratios were calculated for each condition (22°C-other = 1.26, σ = 0.15, *n* = 37,101 pairs; 22°C-swarm = 1.41, σ = 0.14, *n* = 516,198 pairs; 28°C-other = 1.38 σ = 0.20, *n* = 1,040,855 pairs; 28°C-swarm = 1.53, σ = 0.16, *n* = 4,864,000 pairs). The 22°C data were pooled across two independent experiments.

These findings show that the flight tones of male *Anopheles* can assume distinct frequency states. For the vast part of the day, they occupy a baseline state characterized by low frequencies. Around sunset, flight tones increase in frequency and move to a swarming state; after detection of a female-like flight tone, they finally raise their frequencies even higher and enter an activated state. The finding that these three states are centered on a male/female flight tone ratio of 1.5 (baseline, 1.38; swarming, 1.53; and activated, 1.62) is interesting and relevant. For a two-tone, and distortion-based, hearing system such as that of mosquitoes, an interval ratio of 1.5 (also called the perfect fifth in music theory) constitutes a singularity. This results from two low-frequency distortion products, the quadratic distortion product (or “difference tone”), *f*_2_ − *f*_1_, and the cubic distortion product, *2f*_1_ *- f*_2_ becoming numerically identical at a (*f*_2_*:f*_1_) ratio of 1.5, thus creating a “super distortion” (Fig. 3B).

Mosquito flight tones have been reported to vary with ambient temperature (*24*). We tested the daily distribution of flight tones also at 22°C and observed the same phenomena. At swarm time, the flight tone ratio was 1.41 ± 0.14 but dropped to 1.26 ± 0.15 for the rest of the day (Fig. 3B), and as was the case at 28°C, flight tone changes were restricted to males.

At 22°C, we could now also directly compare the frequency response function (resolution Δ*f* = 15 Hz) of the males’ nerves to the spectral bandwidth of distortion products generated by the observed flight tones. In all ears tested, compound nerve responses to sinusoidal flagellar oscillations were limited to frequencies between ~65 and ~400 Hz, with a plateau of maximal responses occurring between ~150 and ~300 Hz (Fig. 3A and fig. S5). Male ears can assume two states, a quiescent (baseline) state and a state of self-sustained oscillations (SSOs) (*16*). In the baseline state, no ear tested showed any response to frequencies >450 Hz (fig. S5). Higher frequency responses have been reported though (*21*, *25*–*27*) and also occurred in our recordings. All of these, however, were linked to SSOs. In the absence of another tonal component (a second tone or an SSO), which contributes to the production of audible distortion, no response to higher frequencies occurred. Comparing the sensitivity of male *Anopheles* nerves to the distortion products available to their ears at swarm time, with those distortion products occurring during the rest of the day, shows how the male-specific flight tone increase harvests female audibility by increasing the overlap between cubic and quadratic distortion products (Fig. 3C).

### Harmonic convergence events are an epiphenomenon of flight tone variance

Our data show how daily modulations of flight tone changes in male *Anopheles* adjust, and optimize, the audibility of females, specifically at swarming time. These modulations are centered on a male/female flight tone ratio of 1.5, which also constitutes an important theoretical optimum. All our data were gathered from separately kept males and females, precluding any interactions between the sexes. We thus wondered whether the previously reported phenomenon of harmonic convergence was an epiphenomenon of the observed daily variations of male/female flight tone ratios around a center value of 1.5 and not a signature of an acoustic interaction, or communication, between male and female mosquitoes during paired flight (for details of our analyses, see Annex 1 in the Supplementary Materials).

**Fig. 4.**
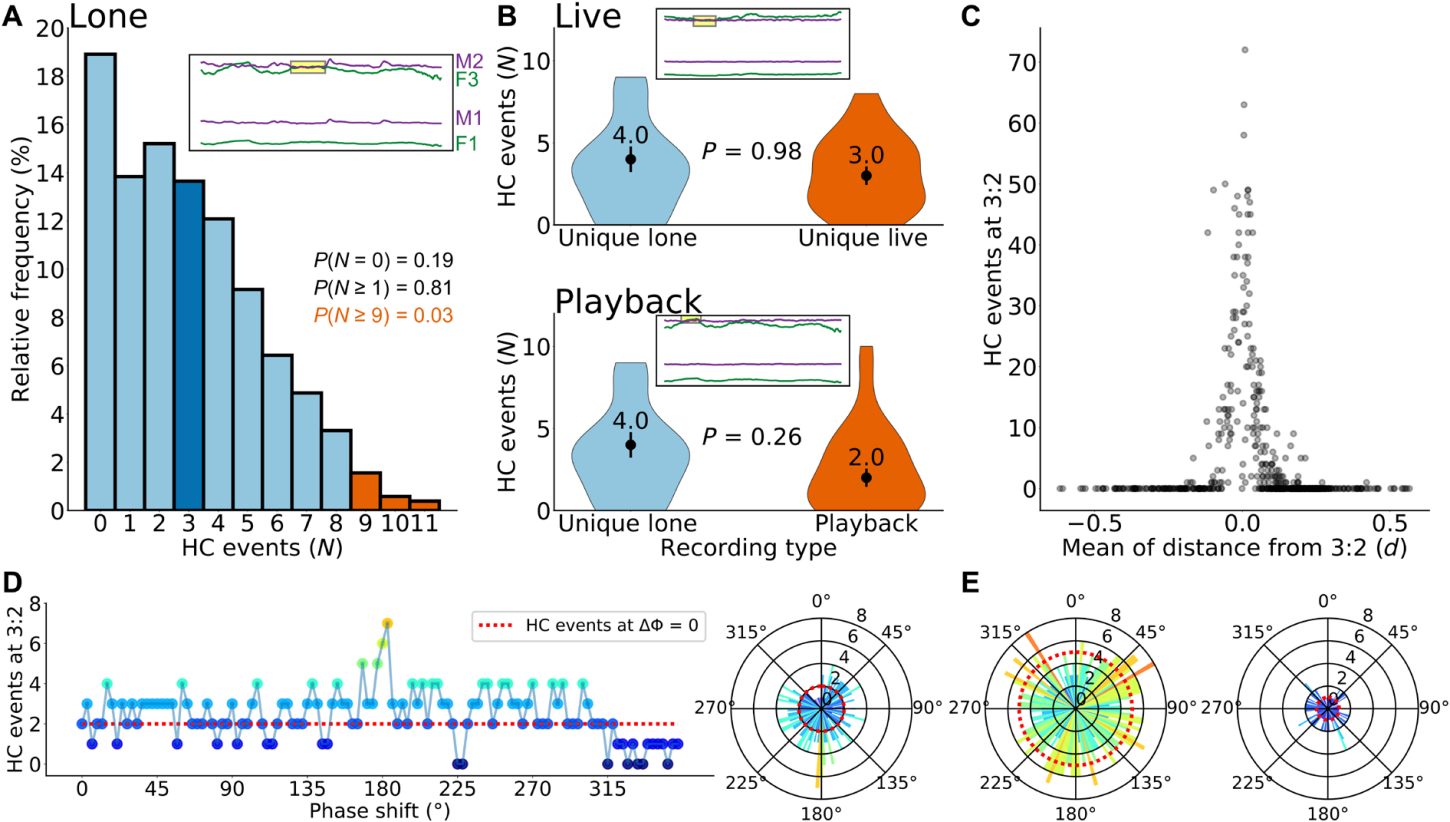
Statistical perusal of harmonic convergence [dataset from (*22*)]. (**A**) The relative frequency distribution of harmonic convergence (HC) event numbers (*N*) from 513 virtual (“lone”) pairs (1-min flights) served as reference for significance analyses in real (“live”) pairs. To have a probability *P* < 0.05 of having occurred by chance, a live pair must exhibit at least nine convergence events. Values below nine are statistically unremarkable (*P* > 0.05). The average convergence event number (see figs. S14 and S15) for both lone and live pairs is ~3. Inset: Harmonic convergence event for a lone pair (M1, male fundamental; F1, female fundamental; M2, male second harmonic; F3, female third harmonic). (**B**) Violin plots of convergence event numbers in live pairs (top) and playback pairs (bottom) compared to convergence event numbers in lone pairs. No statistical differences are observed. Inset: Harmonic convergence event for a live (top) and a playback (bottom) pair. (**C**) For each mosquito pair, convergence events at a given ratio depend on the pair’s mean distance (*d*) from that ratio. Here, *d* is calculated as mean of the absolute distances of a pair’s instantaneous flight tone ratios (over the 1-min flight) from the given harmonic ratio (here, 3:2). The sharpness of the distribution peak highlights the extreme noise sensitivity of harmonic convergence calculations. Pairs whose flight tone ratios are accidentally close to the harmonic convergence ratio produce convergence events by mere chance. Pairs whose flight tones are accidentally far from the harmonic convergence ratio will not produce convergence events at all. (**D**) Left: Linear plot showing convergence event numbers for a real (live) pair of tethered-flying mosquitoes if the circularized time traces of their flight tones are phase-shifted against each other (phase shift, ΔΦ, 0° is the real-time pairing). Right: Polar plot for the same data. (**E**) Polar plots of two other real (live) pairs. Real-time pairing yields suboptimal convergence numbers for all pairs shown; maxima of harmonic convergence counts are randomly distributed across the phase space.

Harmonic convergence describes the transient (1 to 2 s long) match of the third harmonic of the female flight tone with the second harmonic of the male flight tone (Fig. 4, A and B). At a (male/female) fundamental flight tone ratio of 1.5, harmonic convergence will occur by default, independent of any interaction. We therefore conducted an in-depth analysis of the only existing, extensive, and publicly available, experimental dataset (*22*) that had previously been generated to allow for a statistical perusal of harmonic convergence events in *Aedes aegypti* mosquitoes (Fig. 4, figs. S9 to S16, and Annex 1). We found that the number of harmonic convergence events between a specific male-female pair was only a function of their respective median flight tones, more specifically of the distance (or proximity) of the ratio of their median flight tones to a chosen harmonic convergence ratio (table S1), e.g., the ratio of 1.5. The closer a particular pair (virtual or real) was to the 1.5 ratio, the more harmonic convergence events occurred by mere chance (Fig. 4C and Annex 1). Harmonic convergence events were not enriched in real pairs as compared to virtual pairs (composed of pairs chosen randomly from pools of lone-flying males and females). Harmonic convergence events were also not more likely to occur in males exposed to playbacks of female flight tones (number of convergence events per minute, median ± SE: real/live pairs = 3.00 ± 0.56; virtual/loner pairs = 4.00 ± 0.78; playback = 2.00 ± 0.55).

In summary, harmonic convergence events between two given time traces of mosquito flight tones are the result of random overlaps; the resulting number of convergence events depends on the statistical properties of, and interrelation between, the two respective flight tone distributions. The flight tone distributions are independent of each other and not, as one would expect if any interaction or communication would occur, temporally coupled. This fact can be illustrated best by manipulating the phase relation between the two time traces of a real (live) pair (Fig. 4, D and E). Communication or interactions between two partners occur in real time. This is not trivial. In contrast to the real-time coupling of a real (live) pair, the phase relation of the flight tone traces within a pair that was randomly assembled from a library of virtual (lone-flying) mosquitoes is arbitrary. If harmonic convergence events were the result of an interaction or communication, the real-time coupling (i.e., a phase shift of 0°) should constitute an optimality and yield a higher harmonic convergence count than all other possible (and artificial) phase relations of that pair. If, however, harmonic convergence events only arose by chance, then the real-time coupling would just be one arbitrary phase relation among all possible other phase relations of that pair. The harmonic convergence count at real-time pairing should not be significantly different from any other phase relation of that pair.

To test this, we “circularized” the 1-min-long flight tone traces of real pairs from the published dataset (*22*) by “gluing” their beginnings to their ends. For each real pair, this created two circular traces, which could be rotated against each other, thereby creating arbitrary phase shifts between them. As Fig. 4 (D and E) illustrates, the real-time pairing (that means a phase shift of 0°) does not constitute an optimality within live (real) pairs of mosquitoes. Harmonic convergence count maxima are widely distributed across the phase space, as expected for a noninteractive and random-driven process.

We also tested whether flight tones of lone-flying mosquitoes (males and females) were in any other way different from those of flying in pairs (see Annex 1, sections S1.8 and S1.9, and tables S2 and S3), but we found no significant differences in flight tone frequency or variance; all tested cohorts were statistically indistinguishable from each other. In summary, there is no evidence for acoustic interaction between the sexes; all occurring harmonic convergence events were sufficiently explained by chance. This was true for both males and females and for all other harmonic convergence ratios suggested (on this point, see also the section on “phonotypes” below).

### Mosquito flight tone phonotypes

**Fig. 5.**
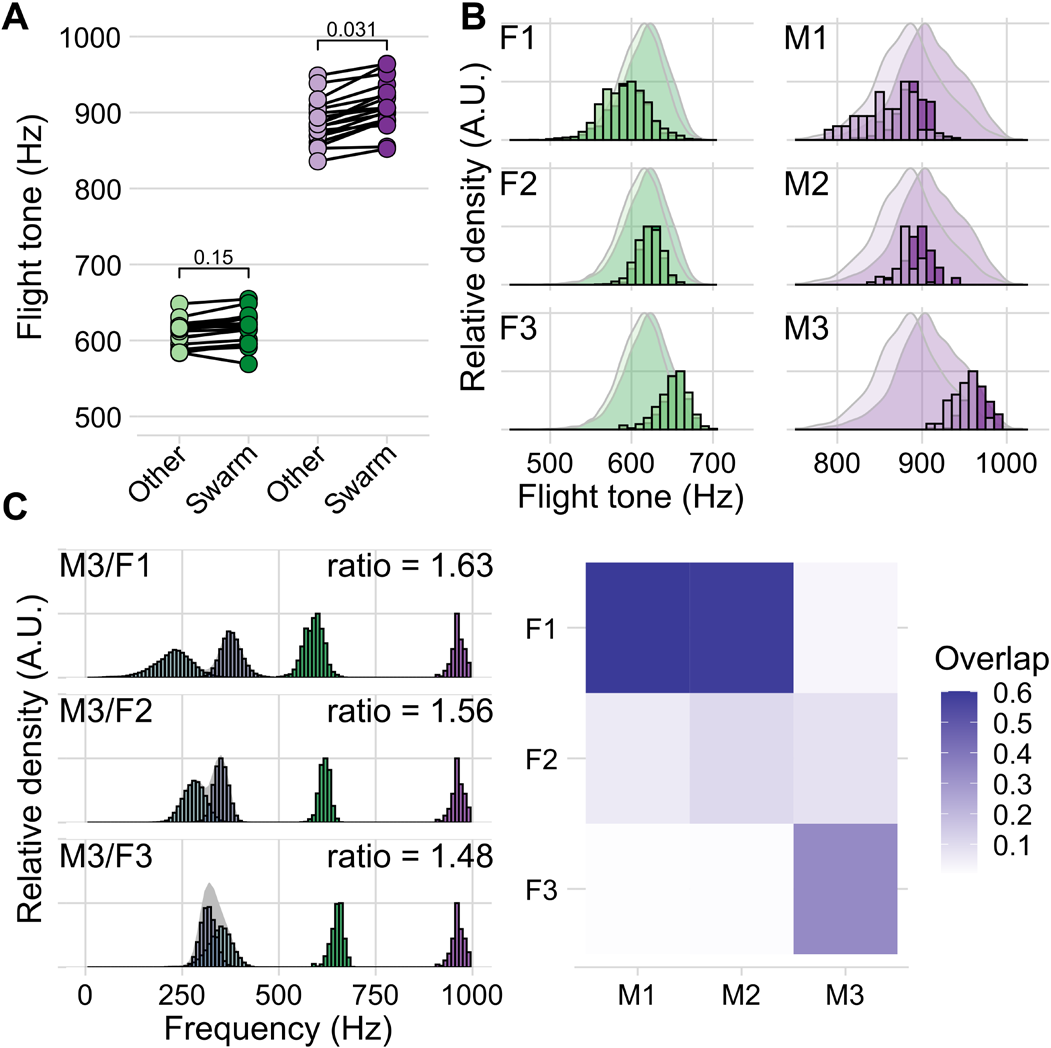
Single mosquito phonotype analysis of individually housed mosquitoes in a custom-made 5 cm–by–5 cm–by–5 cm mini flight arena. (**A**) Swarm and out-of-swarm (other) flight tones of individual female (green) and male (purple) mosquitoes recorded during 12-hour light/12-hour dark entrainment (with 1-hour simulated dusk and dawn) at 28°C. Points are mean flight tones calculated from all detected flight tones in the designated time window for each individual (female-other = 607 Hz, σ = 17 Hz; female-swarm = 616 Hz, σ = 21 Hz *n* = 18 females; male-other = 883 Hz, σ = 29 Hz; male-swarm = 907 Hz, σ = 32 Hz *n* = 18 males). (**B**) Flight tone distributions of three representative female (F1 to F3) and three representative male (M1 to M3) phonotypes. Bar plots are scaled, binned counts of the individuals flight tones recorded during swarm time (darker color) and out-of-swarm time (lighter color). These are plotted against the population scaled density plots for comparison. (**C**) Distribution of distortion products (cubic, light blue; quadratic, dark blue) that would be produced between M3 (purple) and the three representative females (F1 to F3, green). Average ratio value is calculated for every combination of male and female flight tone for each pair (M3/F1 = 1.63, σ = 0.07, *n* = 65,160 pairs; M3/F2 = 1.56, σ = 0.04, *n* = 33,240 pairs; M3/F3 = 1.48, σ = 0.04, *n* = 41,280 pairs). Heatmap displays the proportion of overlap between the two calculated distortion tone distributions for each representative female/male pair. Both female and male data are pooled from three independent experiments each with six individuals.

We tested whether the swarming-related, and male-specific, flight tone modulation seen in groups would also occur in mosquitoes kept individually. As observed under grouped conditions, the flight tones of individual females did not show significant differences at swarm time as compared to the rest of the day (swarm time = 616 Hz, σ = 21 Hz, other time = 607 Hz, σ = 17 Hz; *n* = 18; *P* = 0.19, Welch two-sample *t* test; Fig. 5A). Individual males, in contrast, showed a significant increase at swarm time (swarm time = 907 Hz, σ = 32 Hz, other time = 883 Hz, σ = 29 Hz; *n* = 18; *P* = 0.037, Welch two-sample *t* test; Fig. 5A). When comparing the flight tone ranges of lone-flying mosquitoes, it was evident that both males and females occupied narrower frequency ranges than the population of all individuals of the respective sex pooled together (Fig. 5B and fig. S6A). Within each sex, the flight tones of individual mosquitoes showed statistically significant differences [one-way analysis of variance (ANOVA), *P* < 2 × 10^−16^ in both males and females]. This is remarkable, and relevant, from an acoustic perspective. The audibility of a given female for a given male depends on the extent of audible distortion products that are generated by the mixing of his own flight tone with the flight tone of that particular female (Fig. 5C, left). For an ideally tuned male auditory nerve (Figs. 3), the audibility score of a specific female can be approximated by calculating the overlap between quadratic and cubic distortion products generated by the two flight tones.

The observed individual differences between male and female flight tones, which partly form discrete, narrowband, and non-overlapping, phonotypes, are bound to lead to individual hearing ranges, which means that some males can hear some females better than others (Fig. 5C, right). For low sample sizes, the existence of narrowband phonotypes also introduces distinct peaks into the histograms of flight tone, or flight tone ratio, distributions (see the Supplementary Materials for details). This “peakiness” of the landscape of male/female flight tone ratios has previously been interpreted as a signature of acoustic interactions and led to the postulation of additional harmonic convergence ratios beyond 1.5 (*22*). Our analyses did not find any statistical evidence supporting such a conclusion; the suggested additional ratio peaks also disappear after averaging across appropriate sample sizes.

## DISCUSSION

### Male control of female audibility

The acoustic chase of flying females is a hallmark of reproductive behavior in male mosquitoes. However, a common, and rather unexpected, feature of males across species is that their auditory nerves are near deaf to the actual flight tones of their conspecific females (Fig. 3B and fig. S5) (*25*, *28*). Females will become audible to males, however, if the nonlinear mixing of male and female flight tones produces audible distortions within the male’s ear. Here, the degree of female audibility depends on the specific interrelation of the two flight tones. We found significant daytime- and state-dependent modulations of flight tones in separately caged males but not in separately caged females. The respective audibility space of the mating swarm is under circadian modulation and controlled by males. The detailed quantitative analysis of this audibility control shows how the males’ flight tone (or wingbeat frequency) selection exploits the boundaries of their hearing ranges (Fig. 3, A and B, and fig. S5), revealing a close coupling between the ranges of mosquito wingbeat frequencies and the frequency response functions of their flagellar ears.

Theoretical considerations predict an optimality of distortions to occur around a (male/female) frequency ratio of 1.5. At this optimal ratio, two different distortion products (quadratic and cubic) will summate and produce a super distortion at a frequency equal to half the female’s flight tone (*f*_1_/2). The mosquito auditory nerve seems prepared for these settings; simply dividing the recorded female flight tones by 2 provides a good estimate for the center frequency of male auditory nerve responses (see dotted line in Fig. 3). Multiplying the response plateau of the male nerve by 2, in turn, can predict the distribution width of female flight tones. Notably, while *Anopheles* males optimize their flight tones for female detection in the swarming state, a residual audibility of females is maintained in the baseline state (Fig. 3). The here relevant evolutionary pressures on flight tone selection merit further exploration. The wingbeat frequency upshift that associates with the activated state (i.e., after hearing a female) seems to move the males away from the optimal ratio to female flight tones. This could indicate that during the copulatory chase, males sacrifice female audibility for flight speed. An alternative explanation might be that males shift their flight tones to be less audible to the female, thus flying in “stealth mode.” It could, lastly, be that, during the actual chase, females show a wingbeat frequency upshift themselves (preliminary data from fig. S8 are consistent with this hypothesis), which may also be associated with an increase in flight speed; this might restore the optimal 1.5 ratio and render the females not only more audible again (fig. S8) but also faster and thus more difficult to catch. Whatever the ultimate reason, the observed flight tone changes are bound to affect the mutual audibility in a hearing system, where tonal changes of ~50 Hz can reduce responses by >60% (*29*).

Vast parts of the acoustic ecology of mosquitoes appear to be related to their unique mechanisms of audibility generation. For a mosquito, hearing starts with two inaudible tones; both male and female flight tones evoke mechanical oscillations of the flagellar sound receiver, but these are too high in frequency to elicit responses in the mosquitoes’ auditory nerves. One could thus state that for mosquitoes, their own flight tones constitute “ultrasound.” When stimulated with two tones, however (e.g., male and female flight tone), the nonlinear flagellar receiver produces mathematically predictable distortion products. These distortion products are tones in their own right, some of which evoke auditory nerve responses. In effect, mosquitoes thus transpose inaudible, high-frequency tones into audible, low-frequency ones. They do this through a unique division of labor between (i) the tones produced by their wingbeats during flight, (ii) the nonlinear mechanics of their flagellar sound receivers, and (iii) an exquisite sensitivity of their nerves to the predicted low-frequency distortions. At a male/female flight tone ratio of 1.5, this audibility harvest works at an optimum. The overlap of the second harmonic of the male flight tone with the third harmonic of the female flight tone, which inevitably accompanies a ratio of 1.5, is only an epiphenomenon of this optimality. There is no necessity for the mosquitoes to hear these higher harmonics, and current evidence also suggests that they cannot, by themselves, evoke responses in the auditory nerves. It is, in contrast, rather more likely that distortion product–based hearing in mosquitoes has, at least in part, evolved to overcome the high-frequency limits of their auditory nerves.

These general settings hold true for both *A. gambiae* and *A. aegypti* (fig. S4), but distinct differences exist in their behavioral, particularly their circadian, activity patterns (compare Fig. 2 and fig. S4) (*30*). Males of *Anopheles* show a narrow pattern of activity (and phonotactic responsiveness) (Figs. 1 and 2), almost exclusively occurring at swarm time (ZT13, sunset). *Aedes* males, in contrast, display a much wider range of daily activity, although peaks in activity remain at dawn and dusk (fig. S4) (*31*). In alignment with these behavioral patterns, the flight tone–mediated sensitization to female flight tones is restricted to ZT13 in *Anopheles*, whereas males of *Aedes* appear to remain optimally sensitive to female sounds for larger parts of the day (fig. S7).

Flying with higher flight tones, and thus at higher wingbeat frequencies, can be expected to be costly. Any increase in female audibility that males can gain from beating their wings faster must thus be traded off against the corresponding energetic costs. We assume that the differences seen between the two species reflect differential reproductive strategies. *A. gambiae* males mate mostly in large swarms (with hundreds or thousands of males), which only form once a day at dusk. *Aedes* males mate more widely across the day and form only small swarms (with dozens of individuals), which also form at dusk (*30*).

Rather than reflecting acoustic interactions, or communication, between males and females, mosquito flight tone variations are linked to male-specific adjustments of female audibility. Depending on their circadian or behavioral state, males retune the “pitch” of produced distortions, thus modulating female audibility and increasing (or decreasing) the likelihood of reproductive interactions. The fact that individual mosquitoes can assume distinct flight tone states, the global distributions of which remain centered on male/female ratios of 1.5, produces harmonic overlaps between pairs of mosquitoes by mere chance. Most prominently, here, the second harmonic of the male will occasionally overlap with the third harmonic of the female. The variance (i.e., the noise) of flight tone traces is considerable, and both daytime and the behavioral (or physiological) state of the mosquito can change the baseline of flight tone frequencies. These settings inject multiple biases and a substantial amount of noise into every assay probing for harmonic convergence events. All these confounding factors must be strictly controlled for and equally distributed between the experimental cohorts to exclude statistical artifacts. The same recording from an individual real (live) pair can either produce zero or up to seven harmonic convergence events, depending on the phase relation (between male and female flight tone traces) at which it is analyzed (Fig. 4, D and E). A small (<5%) shift in the baseline flight tone frequency (for example, as a result of the inevitably unphysiological nature of the tethering) can let harmonic convergence events drop from counts of several dozens to zero (Fig. 4C). Our analyses could not detect any significant differences between the baseline flight tones of real or virtual pairs in the largest publicly available dataset (*22*). Previously reported differences in harmonic convergence event counts, which have been proposed as an acoustic interaction between males and females (*21*), are either directly explained by this intrinsic noise or indicative of underlying differences in the baseline flight tones. For example, the finding that (i) sons of pairs that showed more harmonic convergence before mating had greater mating success and that (ii) these offspring themselves showed more harmonic convergence before mating (*32*) simply indicates that a 3:2 ratio of flight tones provides audibility and, thus, mating benefits and (iii) that flight tones are heritable traits [also confirmed in (*29*)]. Attempts to use the short-lived harmonic convergence events as an indicator of genetic fitness have been largely inconclusive, but harmonic convergence has been found to be predictive of successful mating attempts (*33*), which is expected as the phonotactic chase that precedes copulation is an acoustically guided male behavior, which will benefit from the male flying at 1.5 times the female flight wingbeat frequency.

Instead of counting arbitrarily (and only vaguely) defined harmonic convergence events, future research can now focus on the audibility changes that flight tone variance (and specific flight tone combinations) result in. These are likely to be quantifiable, and robust, predictors of mating efficiency in mosquitoes.

Particularly intriguing in this context are the possible trade-offs between flying and hearing. It has been reported that the upshift in male wingbeat frequency after playback of female flight tones coincides with an increase in flight speed (*27*, *34*). The males’ phonotactic search thus receives both aerodynamic and acoustic support. The underlying biophysics of these relations are nontrivial. Forcing a simple harmonic oscillator beyond its best frequency leads to a reduction in oscillation amplitude (e.g., here, the wing stroke angle) and thus, everything else equal, a reduction in flight speed. Mosquitoes, however, appear to have evolved a different mechanism of force generation, related to wing rotation (*35*), which enables higher wingbeat frequencies. It is tempting to speculate that this unique mode of operation evolved to partly uncouple the aerodynamic and acoustic roles of mosquito wingbeats.

The existence of individual (male and female) phonotypes, lastly, suggests that female audibility differs across males, with some females being a better “acoustic match” for a given male than others. The population-genetic consequences of such interindividual variance in mating compatibility could be substantial. While the molecular bases for mosquito flight tone frequencies are still largely unknown, recent work has linked first genetic pathways and also demonstrated the behavioral impact of mutant phenotypes (*29*).

The acoustic fitness of males, i.e., their ability to detect a female flight tone, forms a crucial bottleneck for their reproductive success; its correct assessment requires the knowledge, and triangulation, of the spectrotemporal properties of three elements: (i) the males’ own flight tones, (ii) the females’ flight tones, and (iii) the response function of the males’ flagellar ears (both mechanically and neuronally). On the level of individual pairs, this will allow for a quantitative understanding of mate selection (e.g., female choice/male choice). On the population level, this will not only contribute insights into mosquito evolution but also help optimize mosquito mutants for mass release programs (e.g., gene drive). Harmonic convergence events, lastly, emerge as epiphenomena from the rules that govern the mosquitoes’ distortion-centered flight tone variance. Future mosquito research will need to keep at least one eye on distortions to see clear.

## MATERIALS AND METHODS

### Insect rearing

Mosquitoes were reared at 28°C, with 80% following standard protocols. Briefly, eggs laid onto filter paper were floated using 1% (w/v) tonic salt solution (Blagdon Pond Guardian Tonic Salt). Larvae were fed increasing amounts of TetraMin fish flakes (Tetra) and were split to maintain density, with water being replenished as required. Pupae collected daily, were sexed into 50-ml Falcon tubes, and following eclosion, the adults were then transferred to experimental arenas.

### Activity monitoring

Three, single-sex mosquitoes (3 days old) were aspirated into glass activity tubes (125 mm long, 25 mm ⌀) covered at both ends with cotton wool, with one end soaked in 10% sucrose solution and wrapped in parafilm to maintain a food source and humidity. Mosquitoes within a given tube were acoustically isolated from all other mosquitoes. Activity tubes were then loaded in TriKinetics LAM25 locomotion activity monitors inside of a Percival I-30VL environmental chamber. Activity counts were recorded as infrared beam breaks as the mosquito(es) crossed the midline of the tube; counts were binned at 1-min intervals.

Double plotted actograms and activity plots were created using the Rethomics R package (*36*). Free-running periods were calculated using chi-square periodogram analysis to create a sliding window for peak activity analysis.

### Flight tone recordings

For the single-sex swarm cage experiments (for both sexes), upon eclosion, 100 virgin individuals were aspirated into a BugDorm-1 (30 cm by 30 cm by 30 cm) insect rearing cage that was then placed in a Percival I-30VL environmental chamber. Insects were aged to 3 days after eclosion in experimental conditions before data acquisition began. Chamber conditions were set to a constant temperature of 28°C, with 80% RH and a 12-hour light/12-hour dark cycle with a 1-hour ramped light transition period to simulate both dawn and dusk (the onset of each of these transition periods will be referred to as ZT00 and ZT12, respectively). Audio recordings were then taken using the microphone array placed in the middle of the BugDorm; recordings were either performed at specific times of the day or continuously depending on the experiment. Similar experimental conditions were used for the single-cage mosquito phonotype experiments; however in these, mosquito flight tones were recorded from individually housed mosquitoes in custom-made 5 cm–by–5 cm–by–5 cm flight arenas.

### Data acquisition

For swarm recordings, four particle velocity microphones (Knowles NR-23158-000) were arranged in a cube to capture mosquito flight tones from all directions. The cube was attached to a 4-mm ⌀ rod that was fixed in the center of a 27,000-cm^3^ (30 cm by 30 cm by 30 cm) BugDorm-1 insect rearing cage. For individual recordings, a single microphone was fixed in the center of a three-dimensional printed 125 cm^3^ (5 cm by 5 cm by 5 cm) cage. Microphones were then connected to custom-made preamplifiers (The Neuroscience Electronics Lab, Institute of Zoology, University of Cologne, Germany), which were then connected to a CED 1401 (Cambridge Electronic Design) data acquisition interface. Experiments involving artificial tone playback had a small speaker driver near the microphone, which was triggered using the (digital to analog converter) DAC output channels of the CED 1401 device. Audio was recorded at 50 kHz per channel, and flyby events were identified and quantified using an automated pipeline as described below in the “Signal processing” section.

### Signal processing

The automated pipeline initially segments raw recordings into 1-min chunks before processing. Any DC bias was removed before passing the signal through a digital band-pass filter (fourth order, Butterworth design). Corner frequencies were 300 and 1200 Hz, except in experiments where artificial female flight tones were played, in which corner frequencies were 600 and 1200 Hz. A moving average envelope was computed across the fully rectified signal to identify flyby events (i.e., the envelope is >2*rectified local mean). Identified flyby events were then fitted with a sliding general sinusoidal model [(Eq. 1), where *x* is the flight tone in Hz] to extract frequency information. Fits are calculated for a 10-ms sliding window (50% slide) across the entire detected event. Medians of all fits that had an *R*^2^ > 0.9 were calculated to quantify the main frequency component of each flyby event (fig. S17)

y=sin(2πxt)+cos(2πxt) (1)

Despite signal processing efforts, in some experiments, mechanical background noise from the environmental chamber were detected by the pipeline, often observed as regular, short events (~30 ms) across a very narrow frequency band (μ = 653 Hz and σ = 3.7 Hz). Minimum event length was adjusted until no events were detected during the light phase of the experiment, i.e., when *Anopheles* mosquitoes do not exhibit any flight activity. This value was typically set at >30 ms.

### Nerve recording methodology

Four- to 8-day-old mosquitoes were first sedated on ice and mounted on Teflon rods using blue light–cured dental glue. After application of this glue, only the right flagellum was free to move. The rod was then held in place by a micromanipulator on top of a vibration isolation table, with the mosquito orientated to face a laser Doppler vibrometer at a 90° angle.

To provide electrostatic stimulation to the mosquito ear, a charging electrode was inserted into the mosquito and its electrostatic potential was increased to −20 V relative to the ground. Two electrostatic actuators were placed symmetrically around the flagellum to enable “push and pull” electrostatic stimulation. A recording electrode was then inserted at the base of the right pedicel so that recordings could be made of compound antennal nerve responses to stimulation. Flagellar displacements resulting from stimulation were simultaneously recorded with electrophysiological activity using the vibrometer. At both the beginning and end of the stimulation, free fluctuation recordings were taken of the flagellum to test for changes in auditory capabilities during the experiment.

Stimulation came in the form of monofrequent pure tones (sine waves) played sequentially from 15 to 695 Hz in 10-Hz intervals. Each stimulus playback lasted 2.5 s, followed by a further 2.5 s of silence before the next stimulus began. Each stimulus frequency was played five times.

Nerve data were analyzed using a custom MATLAB script. A DC remove was applied to the data before the response magnitude at the median best frequency of the nerve response was calculated from its power spectrum. The median best frequency of the nerve was defined as the frequency at which the nerve response magnitude was greatest. In total, 8 *A. aegypti* females, 10 *A. aegypti* males, 7 *A. gambiae* females, 7 *A. gambiae* males, 8 *Culex quinquefasciatus* females, and 8 *C. quinquefasciatus* males were included in the final analysis.

### Data analysis

Activity and flight tone data analysis was performed using R 4.0.3 using a number of published packages from the comprehensive R archive network (CRAN) and custom scripts. The signal processing pipeline is available from https://github.com/jaspwn/simbaR. Electrophysiology data analysis was performed using MATLAB using packages and custom scripts.

## Supplementary Materials

### This PDF file includes:

Figs. S1 to S17 Tables S1 to S3 Annex 1: Statistical analysis of harmonic convergence events [dataset from (*22*)] including figs. S9 to S16 References

## Notes

View/request a protocol for this paper from *Bio-protocol*.
