# Supplementary material for: Hitting the right note at the right time: Circadian control of audibility in Anopheles mosquito mating swarms is mediated by flight tones
Source: Sci Adv. 2022 Jan 12;8(2):eabl4844. doi: 10.1126/sciadv.abl4844 (PMC8754303; doi:10.1126/sciadv.abl4844)
Supplement: Supplementary file 1 — Figs. S1 to S17 Tables S1 to S3 Annex 1: Statistical analysis of harmonic convergence events [dataset from (22)] including figs. S9 to S16 References [file sciadv.abl4844_sm.pdf]

Science Advances
AAAS

Supplementary Materials for

Hitting the right note at the right time: Circadian control of audibility in *Anopheles* mosquito mating swarms is mediated by flight tones

Jason Somers, Marcos Georgiades, Matthew P. Su, Judit Bagi, Marta Andrés, Alexandros Alampounti, Gordon Mills, Watson Ntabaliba, Sarah J. Moore, Roberta Spaccapelo, Joerg T. Albert*

*Corresponding author. Email: joerg.albert@ucl.ac.uk

Published 12 January 2022, *Sci. Adv.* **8**, eabl4844 (2022)
DOI: 10.1126/sciadv.abl4844

This PDF file includes:

Figs. S1 to S17
Tables S1 to S3
Annex 1: Statistical analysis of harmonic convergence events [dataset from (*22*)] including figs. S9 to S16
References

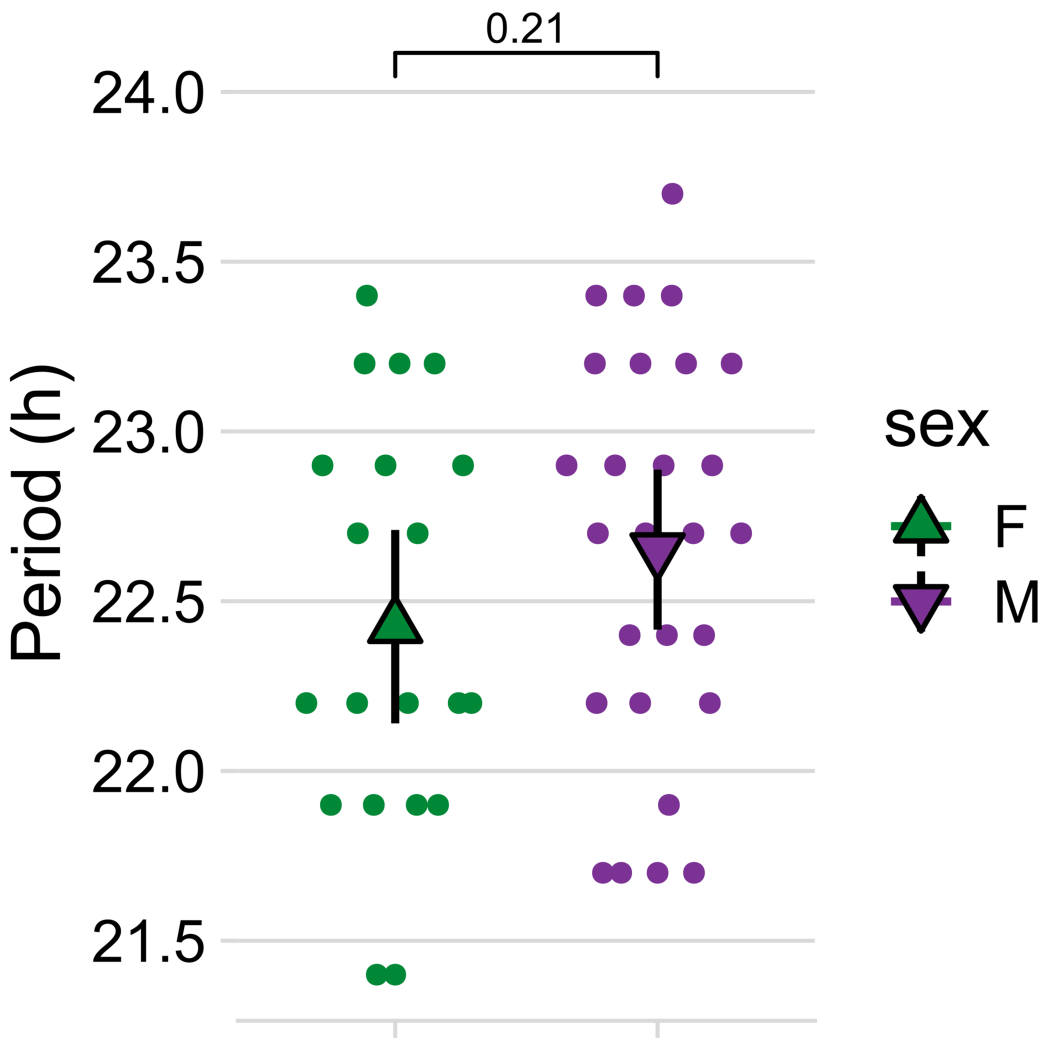

Fig. S1. Period calculations of circadian locomotor pattern. Chi-squared periodogram analysis was used to estimate period length for each group during the free-running phase of the experiment (constant conditions). Points show individual calculated periods and triangles show the group mean for each sex ± S.E.M. (females = 22.43 hours ± 0.14 hours, n = 20; males = 22.65 hours ± 0.11 hours, n = 27). No significant difference in period is observed (p = 0.21, Welch Two Sample t-test).

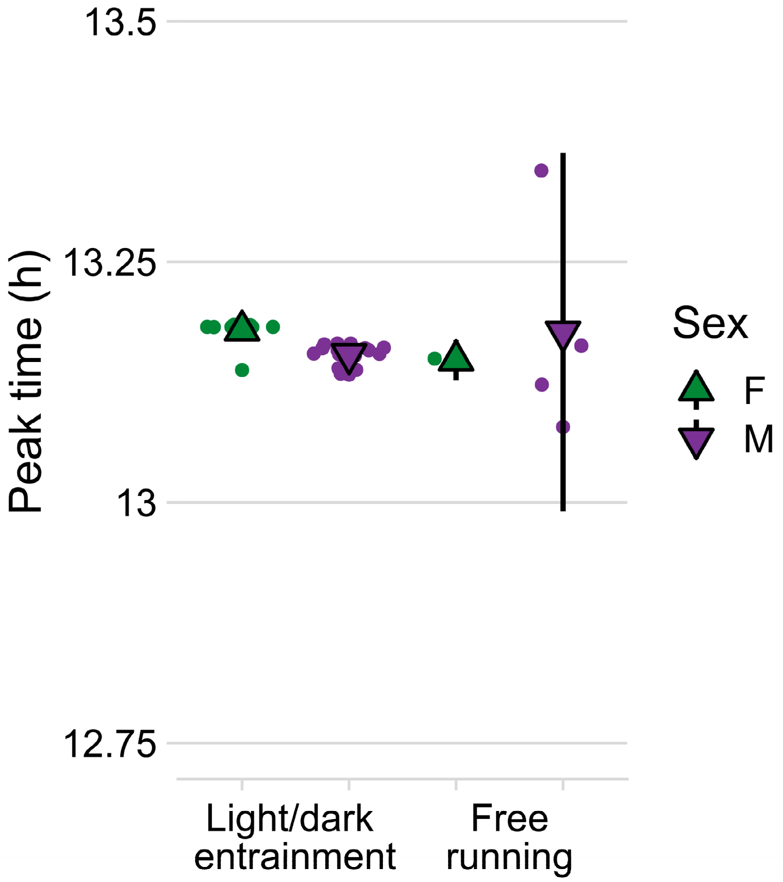

Fig. S2. Daily peak activity of males and females quantified by the maximum number of flyby events past a stationary microphone. Points show daily peak activity and triangles show the group mean for each phase-sex combination ± S.E.M. LD-female = 13.18 h ± 0.004 h S.E.M., n = 12 days; LD-male = 13.15 h ± 0.002 h S.E.M., n = 18 days; FR-female = 13.15 h ± 0.001 h S.E.M., n = 2 days; FR-male = 13.29 h ± 0.05 h S.E.M., n = 4 days.

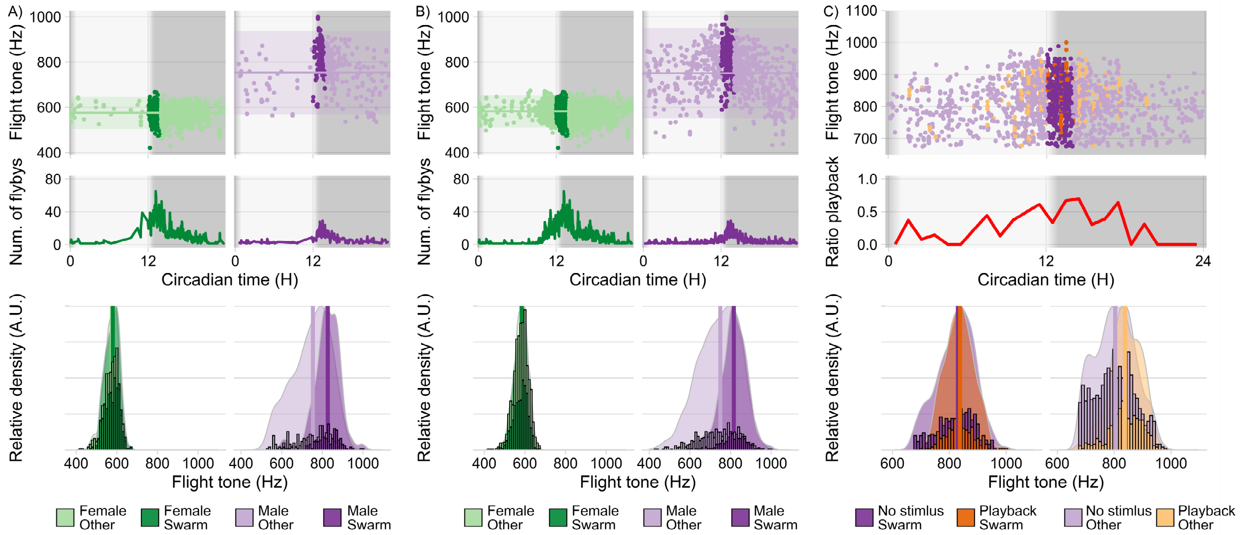

Fig. S3. Acoustic analysis of free-flying populations (~100 single sex individuals) of male and female *Anopheles* mosquitoes housed in 30cm x 30cm x 30cm bug dorm under free-running conditions. A) *Top panels* Individual flight tones recorded from free-flying populations of females and males during the first day of constant darkness at 28°C. Points represent median flight tones of an individual flyby events (see Materials & Methods), darker colors indicate the swarm time period. Solid horizontal line shows the population mean for out-of-swarm flight tones ± 95% C.I. for each sex (female = 577Hz, σ = 36 Hz, n = 989 flight tones; male = 794Hz, σ = 99Hz, n = 395 flight tones). *Middle panels* Line plots showing running averages (window: 5 min) of number of recorded flight tones across the day as a measure of flight activity. *Bottom panels* Distribution of flight tones recorded for each sex across the entrainment days separated by phase – swarm & out-of-swarm (other). Bar plots are binned counts of the individual flight tones and they are plotted against scaled density plots in order to visualize the distribution shift between the swarm and out-of-swarm (other) group. Vertical lines indicate the calculated means for each sex-phase combination (Female-swarm = 579Hz, σ = 34Hz, n = 424 flight tones; Female-other = 576Hz, σ = 36Hz, n = 565 flight tones; Male-swarm = 827Hz, σ = 67Hz, n = 162 flight tones; Male-other = 754Hz, σ = 99Hz, n = 233 flight tones). B) *Top panels* Individual flight tones recorded from free-flying populations of females and males over several days in constant darkness at 28°C. Points represent median flight tones of individual flyby events (see Materials & Methods), darker colors indicate the swarm time period. Solid horizontal line shows the population mean for out-of-swarm flight tones ± 95% C.I. for each sex (female = 580Hz, σ = 36 Hz, n = 1585 flight tones; male = 742Hz, σ = 99Hz, n = 711 flight tones). *Middle panels* Line plots showing running averages (window: 5 min) of number of recorded flight tones across the day as a measure of flight activity. *Bottom panels* Distribution of flight tones recorded for each sex across the entrainment days separated by phase – swarm & out-of-swarm (other). Bar plots are binned counts of the individual flight tones and they are plotted against scaled density plots in order to visualize the distribution shift between the swarm and out-of-swarm (other) group. Vertical lines indicate the calculated means for each sex-phase combination (Female-swarm = 579Hz, σ = 34Hz, n = 762flight tones; Female-other = 580Hz, σ = 36Hz, n = 762 flight tones; Male-swarm = 814Hz, σ = 67Hz, n = 300 flight tones; Male-other = 742Hz, σ = 99Hz, n = 711 flight tones). C) *Top panels* Individual flight tones recorded from populations of free-flying male mosquitoes presented with a 1-minute artificial female flight tone (550Hz) at 30-minute intervals. *Middle panels* Line plot shows ratio between flight tones

recorded during playback of artificial female flight tone and the total number of flight tones within each 30-minute interval ($n_{(playback)}/n_{(total)}$). *Bottom panels* Bar plots are binned counts of individual flight tones; they are plotted against scaled density plots in order to visualize the distribution shift between the playback and no playback group for each experimental phase. Vertical lines indicate the calculated mean for each playback-phase combination (no-swarm = 823Hz, σ = 65Hz, n = 461 flight tones; yes-swarm = 835Hz, σ = 51Hz, n = 223 flight tones; no-other = 803Hz, σ = 69Hz, n = 1136 flight tones; yes-other = 839Hz, σ = 56Hz, n = 383 flight tones). Female data are pooled from 3 independent experiments, male data are pooled from 4 independent experiments and playback data are pooled from 4 independent experiments.

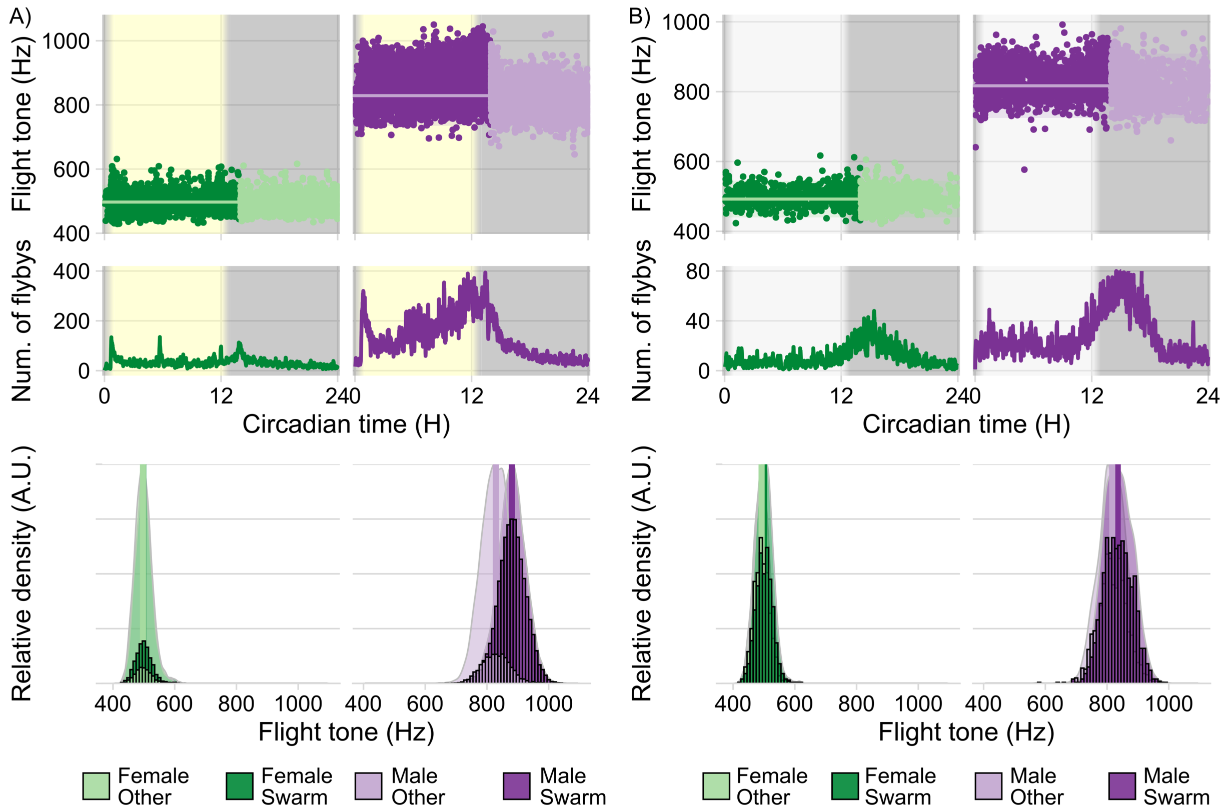

Fig. S4. Acoustic analysis of free-flying populations (~100 single sex individuals) of male and female *Aedes aegypti* mosquitoes housed in 30cm x 30cm x 30cm bug dorms under LD conditions. A) *Top panels* Individual flight tones recorded from free-flying populations of females and males during 12-hour light/dark entrainment (with 1 hour simulated dusk and dawn) at 28°C. Points represent median flight tones of an individual flyby events (see Materials & Methods), darker colors indicate the swarm time period. Solid horizontal line shows the population mean for out-of-swarm flight tones ± 95% C.I. for each sex (female = 498Hz, σ = 27Hz, n = 952 flight tones; male = 830Hz, σ = 47Hz, n = 3202 flight tones). *Middle panels* Line plots showing running averages (window: 5 min) of number of recorded flight tones across the day as a measure of flight activity. *Bottom panels* Distribution of flight tones recorded for each sex across the entrainment days separated by phase – swarm & out-of-swarm (other). Bar plots are binned counts of the individual flight tones and they are plotted against scaled density plots in order to visualize the distribution shift between the swarm and out-of-swarm (other) group. Vertical lines indicate the calculated means for each sex-phase combination (Female-swarm = 499Hz, σ = 27Hz, n = 2374 flight tones; Female-other = 498Hz, σ = 27Hz, n = 952 flight tones; Male-swarm = 880Hz, σ = 45Hz, n = 16220 flight tones; Male-other = 829Hz, σ = 47Hz, n = 3202 flight tones). B) *Top panels* Individual flight tones recorded from free-flying populations of females and males in constant darkness at 28°C. Points represent median flight tones of an individual flyby events (see Materials & Methods), darker colors indicate the swarm time period. Solid horizontal line shows the population mean for out-of-swarm flight tones ± 95% C.I. for each sex (female = 493Hz, σ = 26Hz, n = 784 flight tones; male = 818Hz, σ = 46Hz, n = 1252 flight tones). *Middle panels* Line plots showing running averages (window: 5 min) of number of recorded flight tones across the day as a measure of flight activity. *Bottom panels* Distribution of flight tones recorded for each sex across the entrainment days separated by phase – swarm & out-of-

swarm (other). Bar plots are binned counts of the individual flight tones and they are plotted against scaled density plots in order to visualize the distribution shift between the swarm and out-of-swarm (other) group. Vertical lines indicate the calculated mean for each sex-phase combination (Female-swarm = 500Hz, σ = 27Hz, n = 808 flight tones; Female-other = 493Hz, σ = 26Hz, n = 784 flight tones; Male-swarm = 835Hz, σ = 47Hz, n = 1664 flight tones; Male-other = 818Hz, σ = 46Hz, n = 1252 flight tones). Both female and male data are pooled from 2 independent experiments.

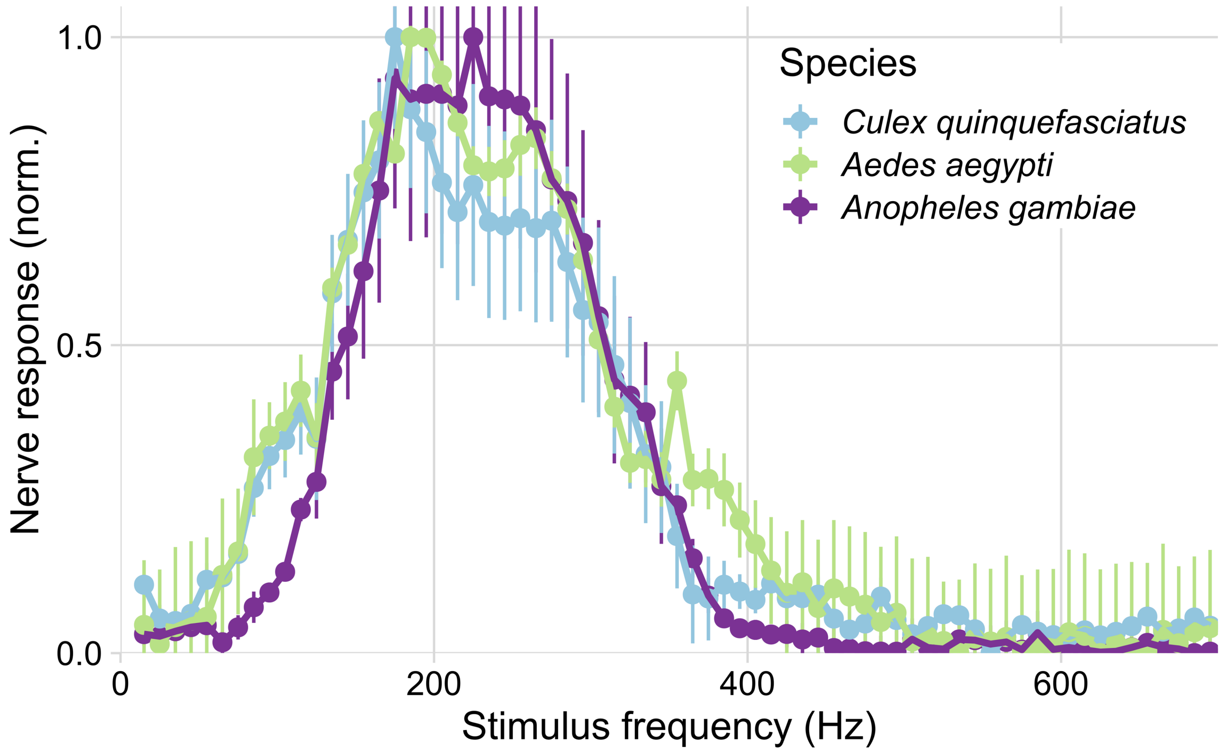

Fig. S5. Normalized male antennal nerve responses to (electrostatic) pure tones are similar across three mosquito species. Sinusoidal (pure tone) stimuli were played sequentially from 15 to 695Hz in 10Hz steps. Nerve responses were similar in all three species and absent for frequencies <50Hz and >400Hz. Responses plateaued between ~150Hz and 300Hz. Points are group medians from male antennal nerves for each species ± S.E.M (n = 8 *Culex quinquefasciatus* males; 10 *Aedes aegypti* males; 7 *Anopheles gambiae* males).

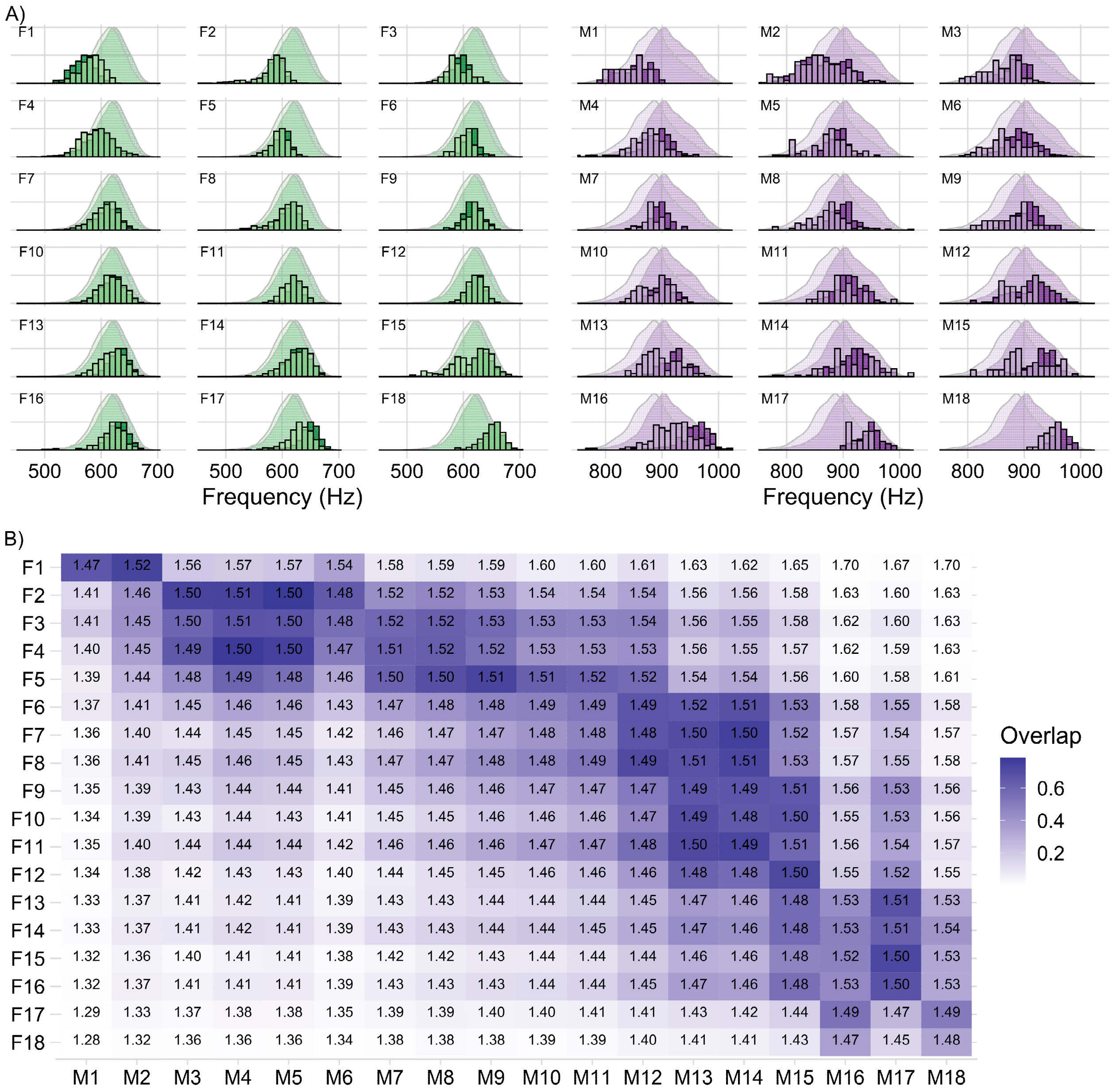

Fig. S6. Flight tone phonotypes and predicted audibility matches between individual male and female *Anopheles* mosquitoes. A) Flight tone distributions of all female (F1-F18) and all male (M1-M18) phonotypes of individually housed *Anopheles* mosquitoes in a custom 5cm x 5cm x 5cm flight arena.. Bar plots are scaled, binned counts of the individuals flight tones recorded during swarm time (darker color) and out-of-swarm time (lighter color). These are plotted against the population scaled density plots for comparison. B) Heatmap displays the proportion of overlap between the two calculated distortion tone distributions for each female/male pair. Inset number is the average ratio value calculated for every combination of female and male flight tone for each pair Both female and male data are pooled from 3 independent experiments each with 6 individuals.

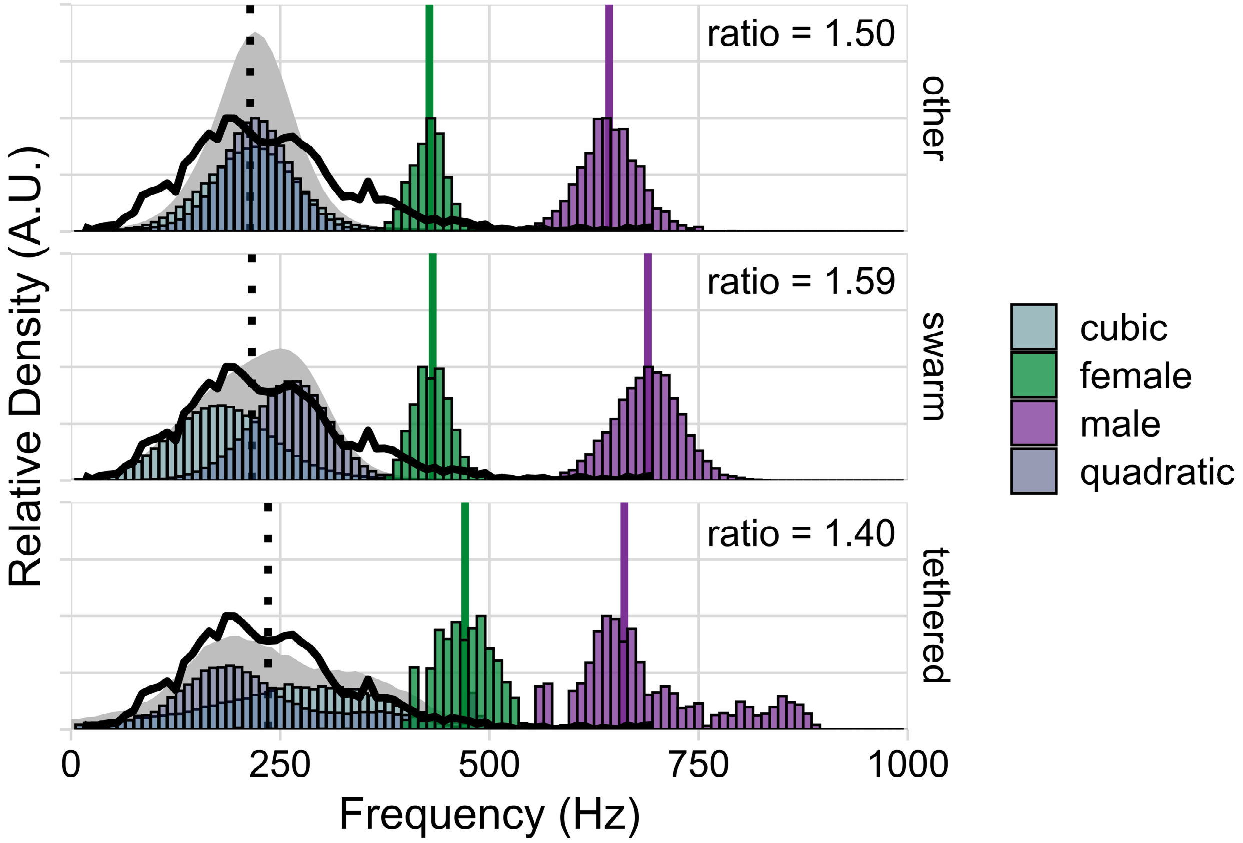

Fig. S7. Predicted distortion products calculated for *Aedes aegypti*. Shown are tethered flight tone data of male and female *Aedes aegypti* (extracted from dataset of ref. (*22*) and collected at ~22°C). For 22°C, we could also match our own nerve recordings (solid black lines) to the predicted distortions. The vertical, dotted black line demarcates an optimum prediction for the center of male nerve responses for a distortion product based hearing system (optimum frequency = 0.5*f1). Note that - in contrast to *Anopheles* (see Fig. 3B) - *Aedes* remains at the optimal (male:female) flight tone ratio of 1.5 for most parts of the day (other); at swarm time, the male-specific wing beat frequency increase lifts the ratio to 1.59 (reminiscent of the 'activated' state in *Anopheles*); during tethered flight, in turn, the average ratio drops to 1.4. The colored vertical lines indicate the calculated mean flight tone for each sex (other: females = 428Hz, σ = 28Hz, n = 670 flight tones; males = 643Hz, σ = 36Hz, n = 1644 flight tones; swarm: females = 432Hz, σ = 27Hz, n = 2243 flight tones; males = 689Hz, σ = 41Hz, n = 9704 flight tones; tethered: females = 471Hz, σ = 36Hz, n = 2280 flight tones; males = 661 Hz, σ = 92Hz, n = 3237 flight tones). Average ratio values are calculated between these medians. Both female and male data are pooled from 2 independent experiments.

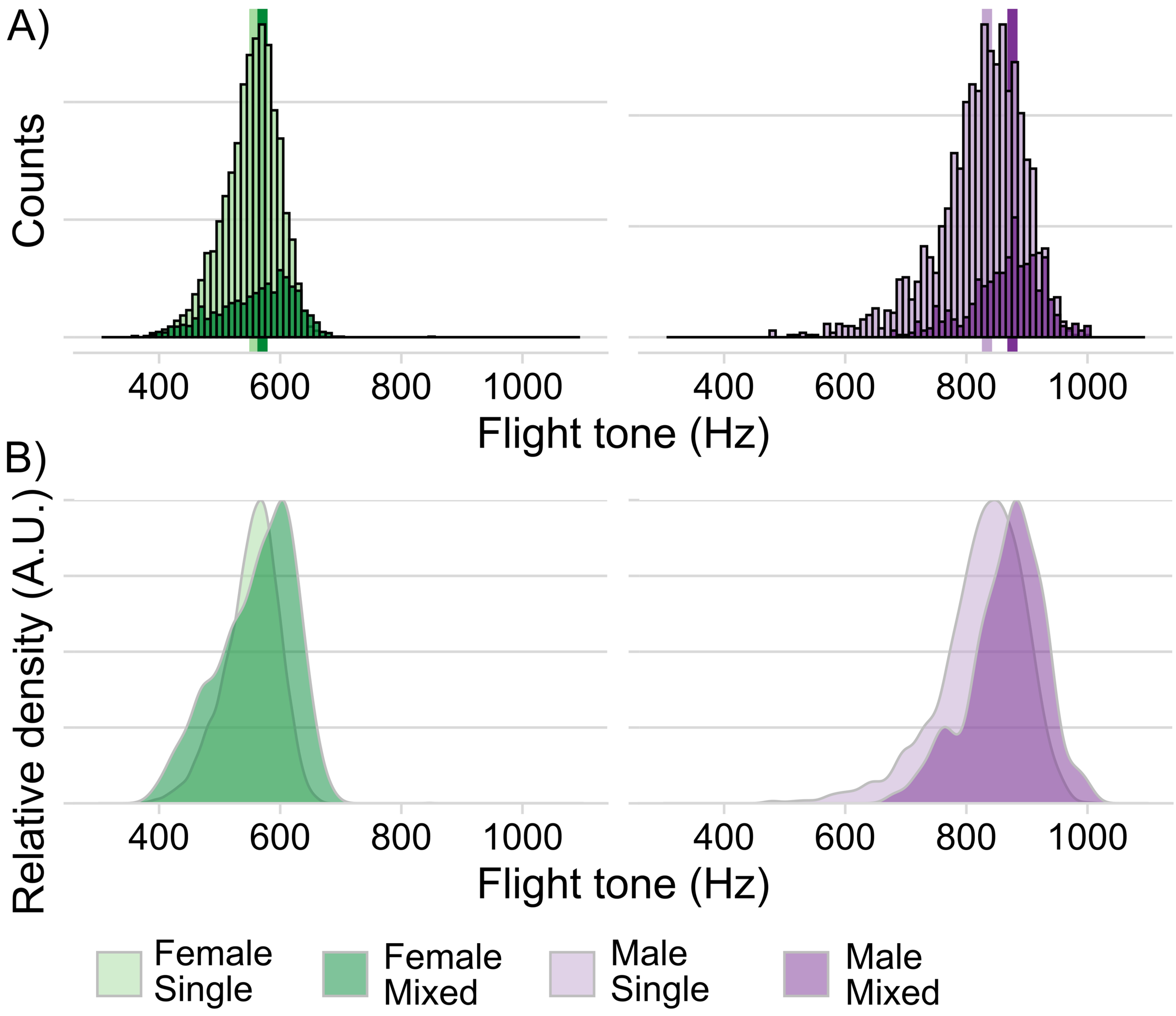

Fig. S8. Comparison of flight tones between single-sex and mixed-sex swarm cages of *Anopheles* mosquitoes. Flight tones (female, green; male, purple) were recorded during 12-hour/12-hour light/dark entrainment (1-hour dusk/dawn transients) at 28°C. A) Bar plots are binned counts of individual flight tones scaled for visibility. A change in distribution can be observed for both sexes between single sex and mixed cages. Females appear to skew to higher frequencies but also increase the low frequency the tail of the distribution. Male distributions shift to higher frequencies with no apparent change in shape. Note that due to the apparent female frequency upshift in the mixed-cages, the male:female flight tone ratio would remain close to the optimum of 1.5. Vertical lines indicate the calculated means for each sex-condition combination (Female-single = 557Hz, σ = 45Hz, n = 5379 flight tones; Female-mixed = 570Hz, σ = 63Hz, n = 1422 flight tones; Male-single = 834Hz, σ = 73Hz, n = 2044 flight tones; Male-mixed = 876 Hz, σ = 61Hz, n = 522 flight tones). Female single sex data are pooled from 3 independent experiments, male single sex data are pooled from 4 independent experiments and mixed cage data are pooled from 2 independent experiments. B) Conversion of flight tone counts to relative density plots to facilitate appreciation of distribution shifts.

Annex 1: Probing harmonic convergence events for statistical signatures of acoustic interaction

1.0 *Synopsis*

When flying in close proximity to each other, male and female mosquitoes have been suggested to interact acoustically by matching their flight tones, either at the level of the fundamental frequency (*37*) or the nearest shared harmonic (*21*). More precisely, for both *Aedes* (*21*), *Anopheles* (*28*) and *Culex* (*19*) mosquitoes, the 2nd harmonic of the male flight tone (M2) was found to converge with the 3rd harmonic of the female flight tone (F3) for short periods of time (<2s). Such a convergence event would correspond to a 3:2 (or 1.5) ratio of the corresponding fundamental flight tones.

We observed a daily shift of fundamental flight tones in male *Anopheles*. Notably, males were caged separately from their female counterparts and the respective cages kept in different incubators, thus ruling out any interactions between the sexes; the males' flight tone shifts were not mirrored by their conspecific females. As a result of the males' flight tone modulations, the corresponding male:female flight tone ratios moved closer to values of ~1.5 during the daily activity peaks of *Anopheles* mosquitoes. We thus wondered if the described 3:2 convergence events simply reflected the random harmonic overlap produced by the males' circadian maintenance of their fundamental flight tones. Rather than signaling an acoustic interaction between male and female, increases in harmonic convergence events would simply be the result of random fluctuations around a given pair's median flight tone ratio, and its (likewise random) proximity to the respective harmonic convergence ratio (e.g. 1.5).

Robust tests of these relations are missing; only a single study (*22*) has explored this question experimentally before and in large enough sample size; they made their data publicly available at https://data.bris.ac.uk/data/dataset/1a44saul2ijj31u0raipvlims6. We conducted an in-depth statistical analysis of the study's data set, and also applied some of the novel approaches introduced by the authors (*22, 38*). In a nutshell, we compared unique *real* (live) pairs to unique *virtual* (lone) pairs, making a deliberate attempt to reduce any distortions that arise from reusing individual pairs. We found that random overlaps are sufficient to explain both the spectral nature, and respective probability, of harmonic convergence events. An assumption of acoustic interaction is neither required nor statistically justified.

1.1 *Aedes aegypti* tethered flight data

The data set from ref. *(22)*, which we analyzed, used *Aedes aegypti* and tethered flight recordings throughout. Our own free-flight experiments confirm that just as in *Anopheles*, *Aedes* males – but not females – show daily (and state-dependent) modulations of their flight tones (Fig. S4 and S7), which include an upshift of flight tone frequencies during swarm time (dusk). Consistent with previous reports, the flight activities of *Aedes* mosquitoes (both males and females) were more evenly spread across the Light:Dark cycle and showed higher flight

activities during the light phase than *Anopheles* (Fig. 2). Probably reflecting this enhanced level of behavioral activity, the male:female flight tone ratio in *Aedes* remained close to ~1.5 for most parts of the day (Fig. S7, top). At swarm time (and driven by a male-specific increase in wing beat frequency) it rose further to values of ~1.59 (Fig. S7, middle). Interestingly, though, analyses of the data from ref. (*22*) reveal a flight tone ratio of only ~1.4 during tethered flight (Fig. S7, bottom). Thus, just as in *Anopheles*, the flight tones of male *Aedes* are state-dependent and the corresponding (male:female) ratios remain close to the theoretical optimum of 1.5. We therefore scrutinized the data set made available by ref. (*22*) to test if harmonic convergence events merely arose by chance.

The specific recordings used for each analysis are stated in the relevant sections. Our investigation focuses on two subsets of the authors' database: (i) Flight tone recordings of *live* male-female pairs,(ii) *lone* males and females, and (iii) *live* male *playback* female 'pairs'. A *live* (or real) pair is defined as a pair of tethered mosquitoes beating their wings in proximity to one another while the signal produced by each of their wingbeats (i.e. the flight tones) is recorded by separate microphones. These data are available at the above stated web resource under the heading 'live_oppsex_pairs'. A lone male or female is defined as a tethered mosquito beating its wings in isolation (isolated from any other mosquito), while its flight tone is recorded by a microphone. Male and female *lone* recordings are also available at the web resource under the heading 'lone_males' and 'lone_females', respectively. A *lone* (or virtual) pair is produced by randomly pairing a lone male with a lone female. A *live* male *playback* female pair is defined as a lone tethered male beating its wings while being stimulated with the playback of a pre-recorded female (live or lone). These data are available at the above stated web resource under the heading 'playback_pairs'.
All flight tone recordings are one minute long and sampled at 40 kHz.

1.2 Flight tone extraction analysis

The flight tones of *Aedes aegypti* produced during tethered flight were extracted following a version of the protocol exemplified in ref. (*38*); here adapted to, and coded in, Python. Briefly: As an initial processing step, the recordings were bandpass filtered (lower cutoff point at 200Hz and higher cutoff point at 1,000Hz). The one-minute long recordings were segmented into non-overlapping windows of length τ = 100ms. Starting with the first segment (i.e. 0 - 100ms) a Fast Fourier Transform (FFT) was then applied to each segment. From the FFT we extracted f_L and f_H, the distances (in Hz) to the left and right of f_o, the most prominent peak in the frequency domain. Here f_L and f_H are given by:

$$f_L = f_o - 50$$

$$f_H = f_o + 50$$

These two limits were then used to supply a bandwidth for bandpass-filtering the segment. The Hilbert transform was subsequently applied to the bandpass-filtered segment to extract

the respective instantaneous frequency of the mosquito's flight tone over that segment. This procedure was repeated for all flight segments, which were then appended together into the instantaneous frequencies of the complete 1-minute long flight. Finally, as described in ref. (*22*), the same piecewise aggregate approximation (PAA) averaging procedure, with window $w = 0.5$ s, was applied to the instantaneous frequencies to reduce noise. The result was a set of 120 frequency points ($w = 0.5$s, resulting in 2 frequency values per second over the entire 1 minute of tethered flight) for each mosquito recording.

1.3 Reconstruction of figure 6a from ref. (*22*)

Forty-three *live* pairs of male-female *Aedes aegypti* were originally used by the authors to generate a histogram of frequency ratios (*4*). Twenty-four of these forty-three pairs were unique pairs (i.e. different male *or* different female), with the remainder re-using individual pairs (i.e. same male *and* same female). For the construction of unique pairs, the original study of ref.(*22*) also repeatedly re-used individual males or females. For example: Unique pair *A* consists of male m_1 and female f_1 and unique pair *B* consists of male m_1 and female f_2. The frequency ratios of the forty-three live pairs were calculated here, in accordance with the authors analysis as follows:

A set of 120 frequencies, $\{f_1, f_2, \ldots, f_{120}\}$, was obtained for each mosquito of a pair by applying the above mentioned PAA averaging procedure over the minute-long flight. If we define $f_{mi,j}$ and $f_{fi,j}$ as the ith frequencies obtained from the male (m) and female (f) of the jth pair. Then the ith instantaneous frequency ratio of this pair is given by:

$$r_{i,j} = \frac{f_{mi,j}}{f_{fi,j}}$$

Resulting in a set of 120 instantaneous frequency ratios for each pair. The $r_{i,j}$ were pooled together to reconstruct the frequency ratio histogram for live mosquito pairs (bin width = 0.01).

Sixty-eight (virtual) pairs, created from *lone* male and *lone* female *Aedes aegypti*, were used to create a frequency ratio histogram in the original analysis of ref. (*22*). These were constructed as follows: The study's database contained data from 27 lone males and 19 lone females. These were combined by the original study to create a total of 513 lone pairs, from which 68 were randomly sampled (the specific identity of these samples is unknown). The original work described in ref. (*22*) then conducted a frequency ratio analysis for these *lone* (virtual) pairs and compared them to the frequency ratio histogram from *live* (real) pairs. For our own analysis, all 513 lone pairs were used to reconstruct the complete frequency ratio histogram for *lone* (virtual) mosquito pairs (bin width same as above) (Fig. S9).

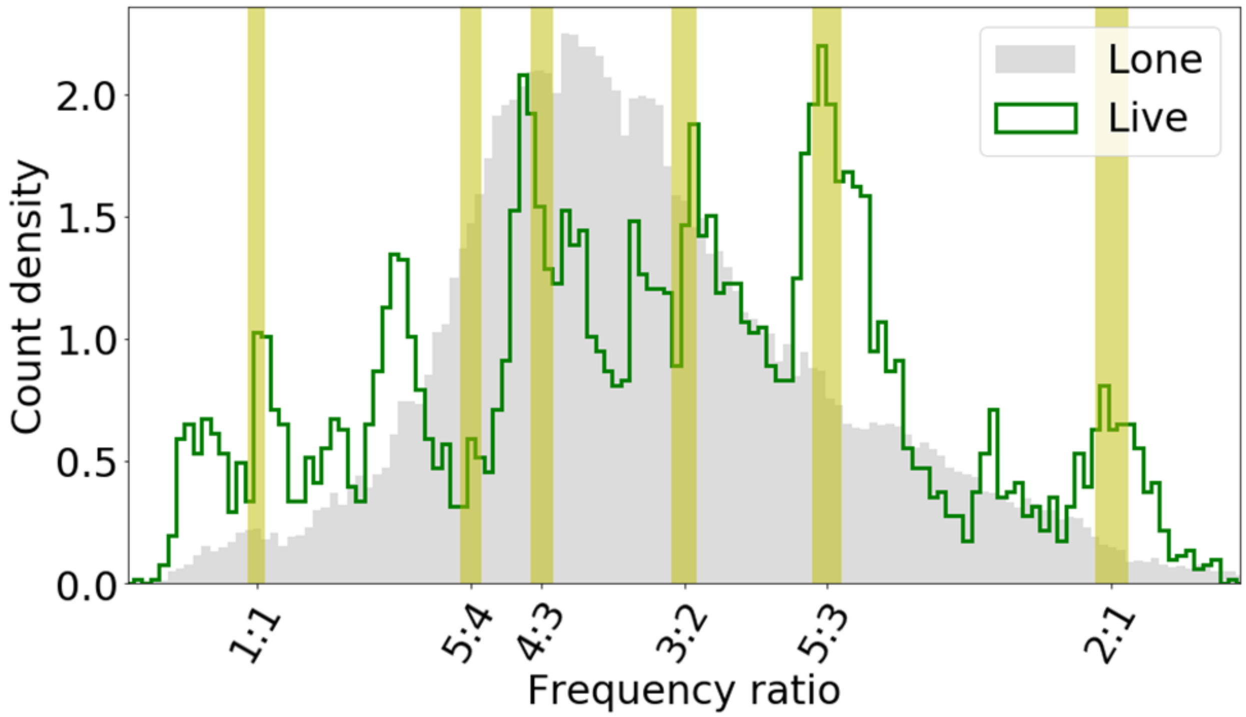

Fig. S9. Reconstructing the landscape of flight tone ratios for *Aedes aegypti: lone vs live pairs*. In a first step, we reconstructed the (male:female) flight tone frequency ratio histogram for tethered flights collected by ref. (*22*) for both live (real, green) and lone (virtual, grey) pairs of *Aedes aegypti* mosquitoes. Forty-three live and 513 lone pairs were used in total. The graph shows a smooth (~unimodal) distribution for lone (virtual) pairs but a peaky (multimodal) distribution for the live (real) pairs, as also reported by the original study (*22*).

Ref. (*22*) compared the frequency ratio histograms of *live* and *lone* pairs, also with those of 34 live male-playback female pairs. These were constructed by stimulating 7 live males with subsets of 12 pre-recorded playbacks of females. We applied our analysis to the 34 pairs (here termed playback) to reconstruct the histogram that includes *live, lone* and *playback* frequency ratio distributions (bin width as above). Note that this is a part reconstruction of the original paper's Figure panel 6b, as it does not include the frequency ratio distribution for live females stimulated with playbacks of males.

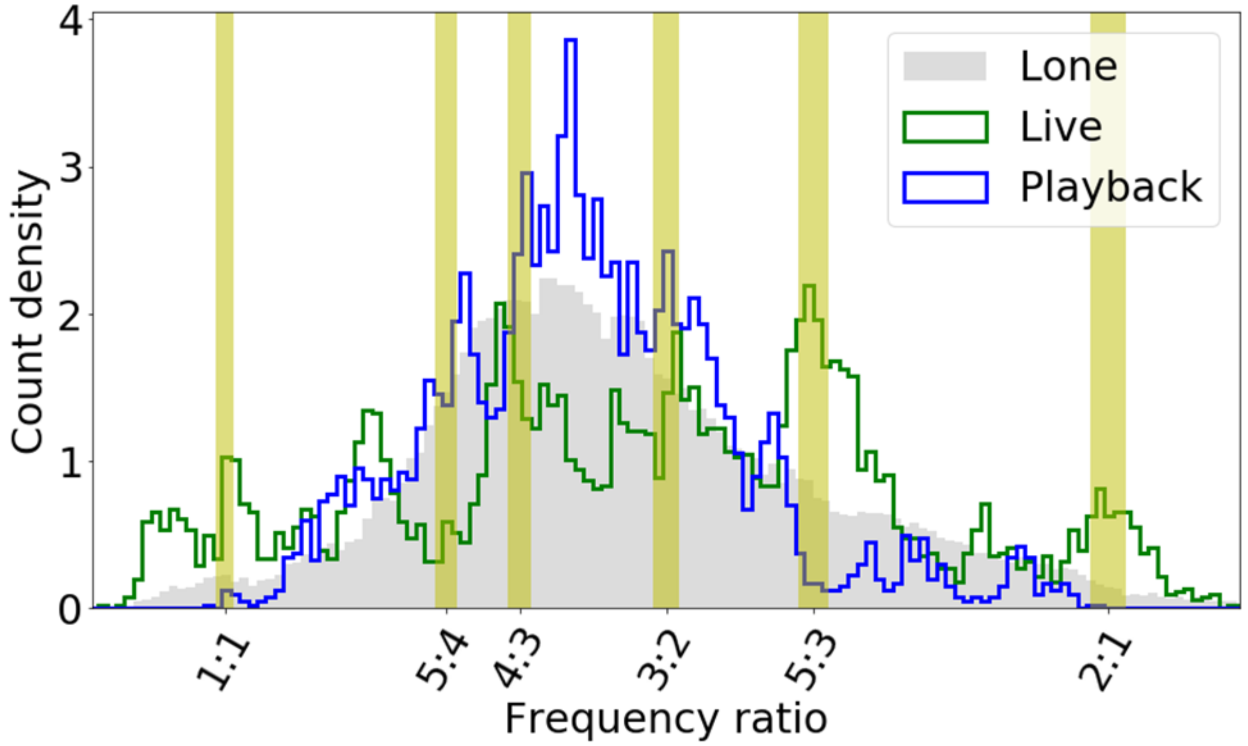

Fig. S10. Reconstructing the landscape of flight tone ratios for *Aedes aegypti: the playback cohort.* Reconstructed (male:female) flight tone frequency ratio histogram for tethered flights collected by ref. (*22*) for live (real, green), lone (virtual, grey), and pairs made of live males and virtual female stimulus (playback, blue). Forty-three live, 513 lone, and 34 playback pairs were used in total.

1.4 Deconstruction of Figure 6a from ref. *(22)*

A key observation made by ref. (*22*) was the difference between (male:female) flight tone frequency ratio histograms from *live* (real) and *lone* (virtual) pairs. While lone pairs showed a smooth, (near unimodal) distribution, *live* (real) pairs showed a multimodal distribution, with clearly identifiable peaks at distinct frequency ratios. To test if these differences, rather than reflecting unique acoustic interactions within live pairs, are caused by the different sample sizes used (*live* n=43, *lone* n=68) pairs, we randomly selected 43 and 68 lone pairings from the total of 513 and used these subsets to produce frequency ratio histograms. Doing so, illustrates how the histogram differences reported by the original study (*22*) between *live* and *lone* pairs (and also confirmed by our own analyses, see *Fig. S9*) can be qualitatively reproduced solely comparing two sets of *lone* pairs (with n=43 and n=68, respectively) (*Fig. S11*).

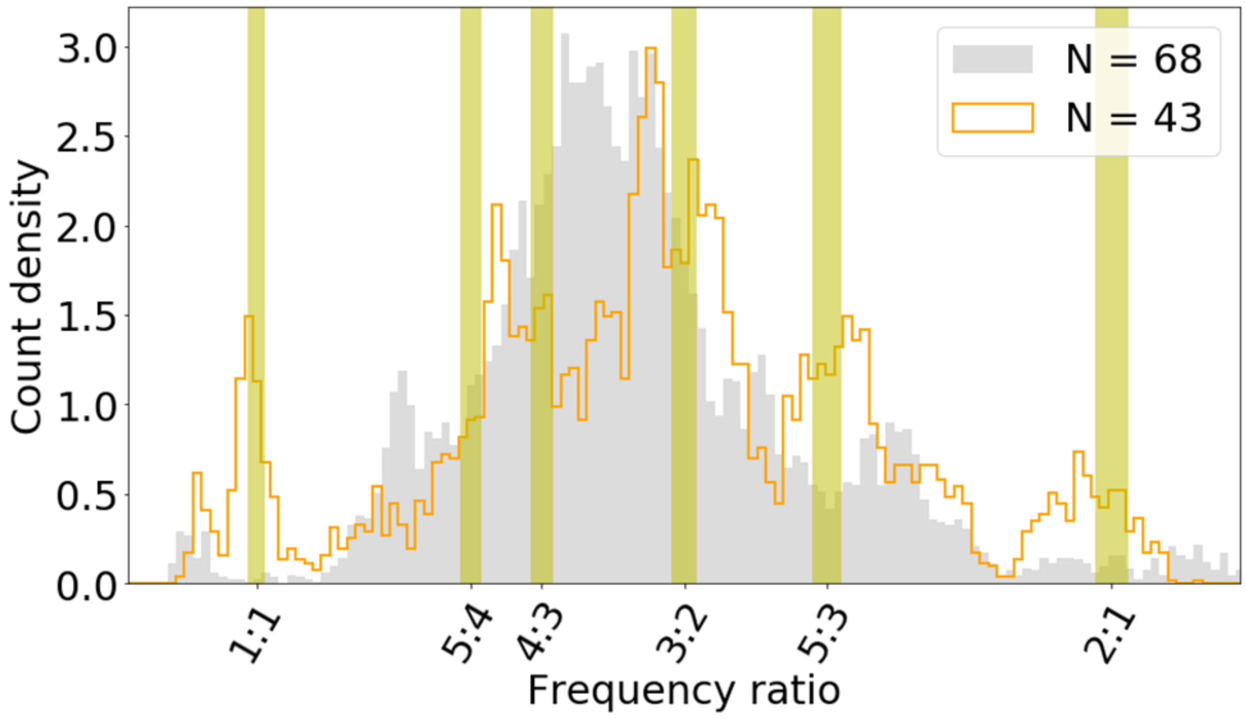

Fig. S11. Reconstructing the landscape of flight tone ratios for *Aedes aegypti: the effects of sample size.* An illustration of how the number of pairs used can affect the shape of the distribution. These are qualitatively similar histograms to those of Figure S11, albeit produced using solely two subsets of lone pairs (with n= 68 and n=43, respectively) randomly selected from the total of 513.

In an additional 'bottom-up' approach we analyzed the frequency - and frequency ratio - distributions of individual mosquitoes – and mosquito pairs from ref. (*22*) to trace the origins of the two different distribution shapes seen on the 'population' level. A look at the individual level shows that each mosquito (both males and females) occupies only a narrow range of flight tone frequencies (*Fig. S12, left*), much narrower than the flight tone distributions of all available samples combined would be. This phenomenon is equivalent to the '*phonotypes'* we observed for individual free-flying *Anopheles* (see Fig. 5 and S6). The narrow frequency ranges of individual males and females translate into sharp and narrow frequency ratios (*Fig. S12, right*), which in turn introduce sharp peaks into the population histograms. For low sample sizes (especially if re-using individual, or individual pairs of, mosquitoes) these distinct ratio peaks cannot be sufficiently averaged out and thus remain visible (as in *Fig. S9-S11* and Figure 6 from ref. (*22*)). The peaky spectral landscape of flight tone ratios reported for live pairs is thus introduced by the phonotypic nature of individual mosquitoes; it does not require, or reflect, any acoustic interaction between male and female. Rather, it is a phenotypic relic of a statistically incomplete, and thus non-representative, population sample.

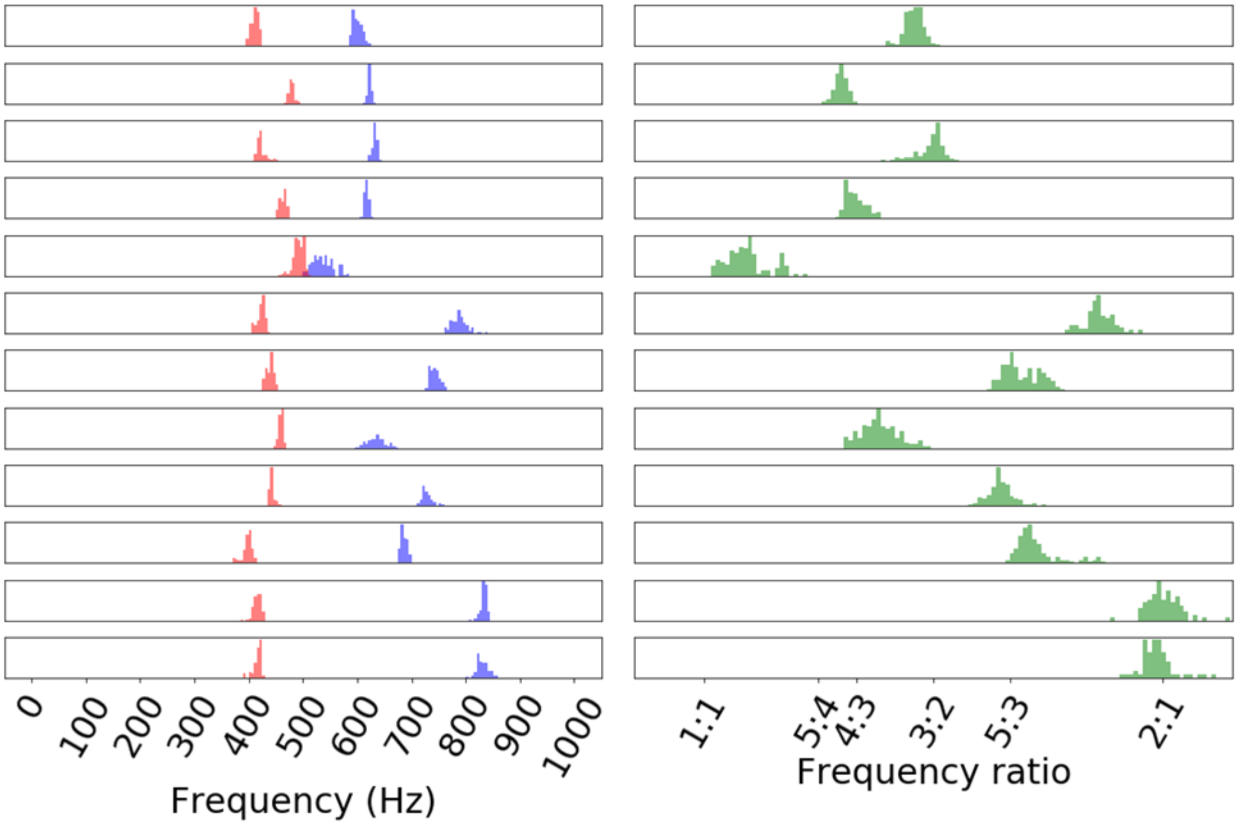

Fig. S12. Examples for the existence of 'phonotypes' in *Aedes aegypti*. Individual mosquitoes, and pairs of mosquitoes, occupy narrow ranges of frequencies, and frequency ratios. These narrow individual distributions introduce distinct peaks when used to calculate population histograms, which will only be averaged out by a sufficiently wide, and sufficiently representative, sampling of the underlying population (data from ref. (*22*)).

1.5 Probability distribution of the number of harmonic convergence events of lone pairs

We now probed if harmonic convergence events were more likely to occur in live pairs. Ref. (*22*) defined a harmonic convergence event as a male:female frequency ratio $r_{i,j}$ (defined above) that meets the following three criteria:

(i) The ratio falls within any of the following ranges: {[0.99 - 1.01], [1.2375 - 1.2625], [1.3199 - 1.3466], [1.485 - 1.515], [1.65 - 1.6833], [1.98 - 2.02]}; that is, it is within ±1% of any of six ratios {1/1, 5/4, 4/3, 3/2, 5/3, 2/1}. These are the ratios that would result from harmonic convergence occurring at the harmonic ratios (male:female) 1:1, 5:4, 4:3, 3:2, 5:3, 2:1.

(ii) The ratio is repeated such that it falls within the respective range for at least 1s (that is, it is repeated in at least two subsequent time windows, given the PAA window of 0.5 s). In other words, the pair maintains harmonic convergence for at least one second.

(iii) the ratio occurs at least 1s after the termination of a preceding harmonic convergence event. That is, there should be a gap of at least one second between any two harmonic convergence occurrences if the second occurrence is to qualify as a harmonic convergence event.

Following these criteria, we calculated the random variable *N*, defined as the number of harmonic convergence events produced by each of the 513 lone pairs. We then calculated the corresponding relative frequency distribution (Fig. S13). Given the large number of samples, this relative frequency distribution approximates the underlying probability distribution and thus served us as reference distribution for our null hypothesis, which would posit that all harmonic convergence events are the result of random frequency overlaps between male and female flight tones. Similarly, the number of harmonic convergence events produced by *live* pairs was determined and the corresponding averages (mean and median) were computed for comparison against the reference distribution (Fig. S14 shows results for all pairings, Fig. S15 for unique pairs only).

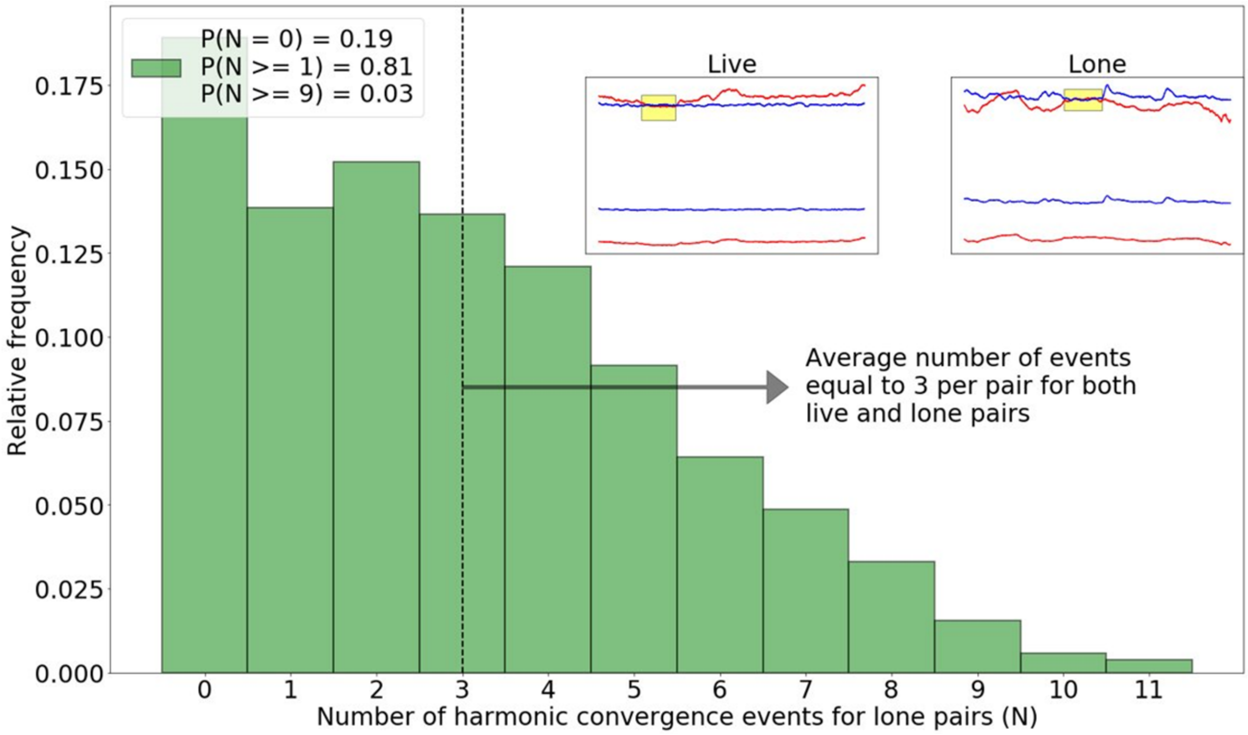

Fig. S13. Relative frequency distribution of the number of harmonic convergence events (N) counted for the 513 lone (virtual) pairs. This distribution serves as reference (null hypothesis) to test if harmonic convergence events are significantly different in live (real) pairs. For an individual harmonic convergence event to be of statistical significance (i.e. to have a probability $p<0.05$ of having occurred by chance), a live pair must exhibit at least 9 harmonic convergence events during the one-minute long flight. Any value below 9 does not constitute a statistically noteworthy event (i.e. it has a probability $p>0.05$ of having occurred by chance). The average number of harmonic convergence events (see also Figs. 4

and S14) for both lone and live pairs is ~ 3. The insets illustrate an example of harmonic convergence at 3:2 for live and lone pairs (blue: male; red: female; shown are fundamentals, the male's 2nd harmonic and the female's 3rd harmonic; harmonic convergence events highlighted in yellow).

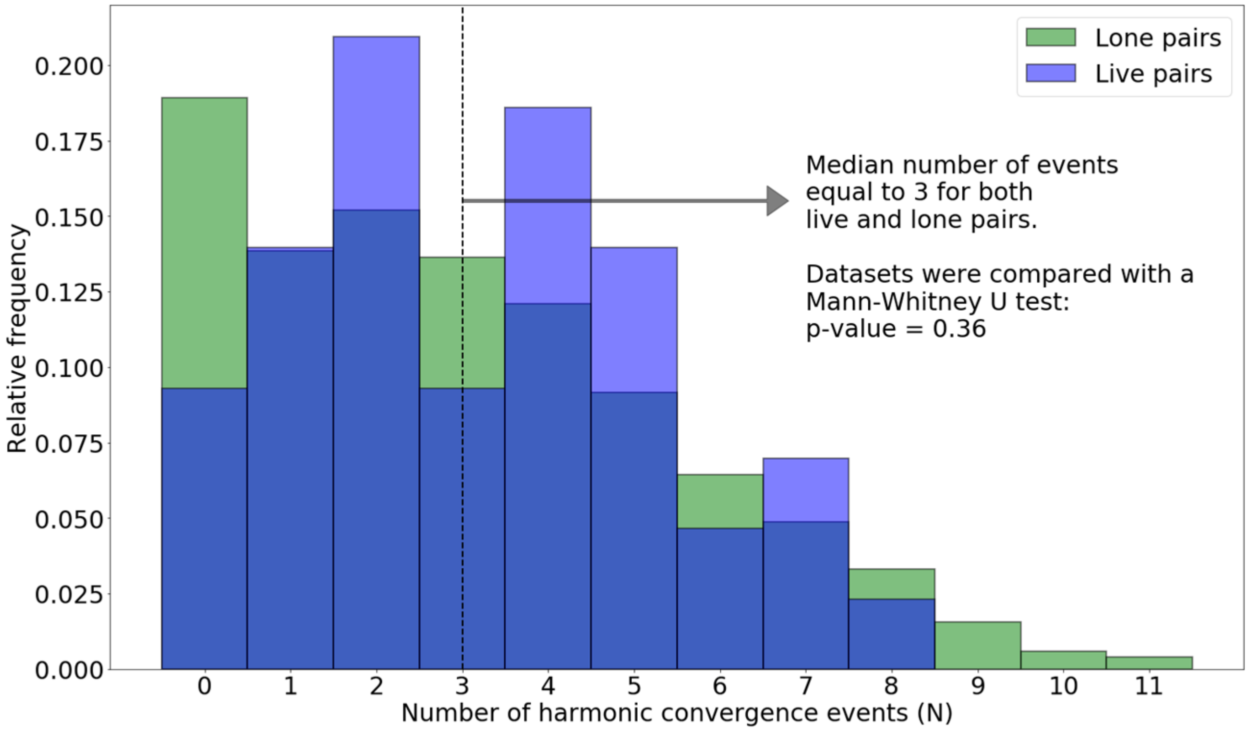

Fig. S14. Superimposed relative frequency distributions of the 513 *lone* (green), and 43 *live* (blue) pairs of mosquitoes show no statistically significant difference. Comparing the distributions of harmonic convergence events between lone (=virtual) and live (=real) pairs shows no statistically significant differences. Indicated in the figure is the result of the comparison of the two datasets that formed the distributions. Test used: Mann-Whitney U test with p-value = 0.36. (for comparison: The Mann-Whitney U test comparison of lone pairs with playback pairs gives a p-value of 0.17.)

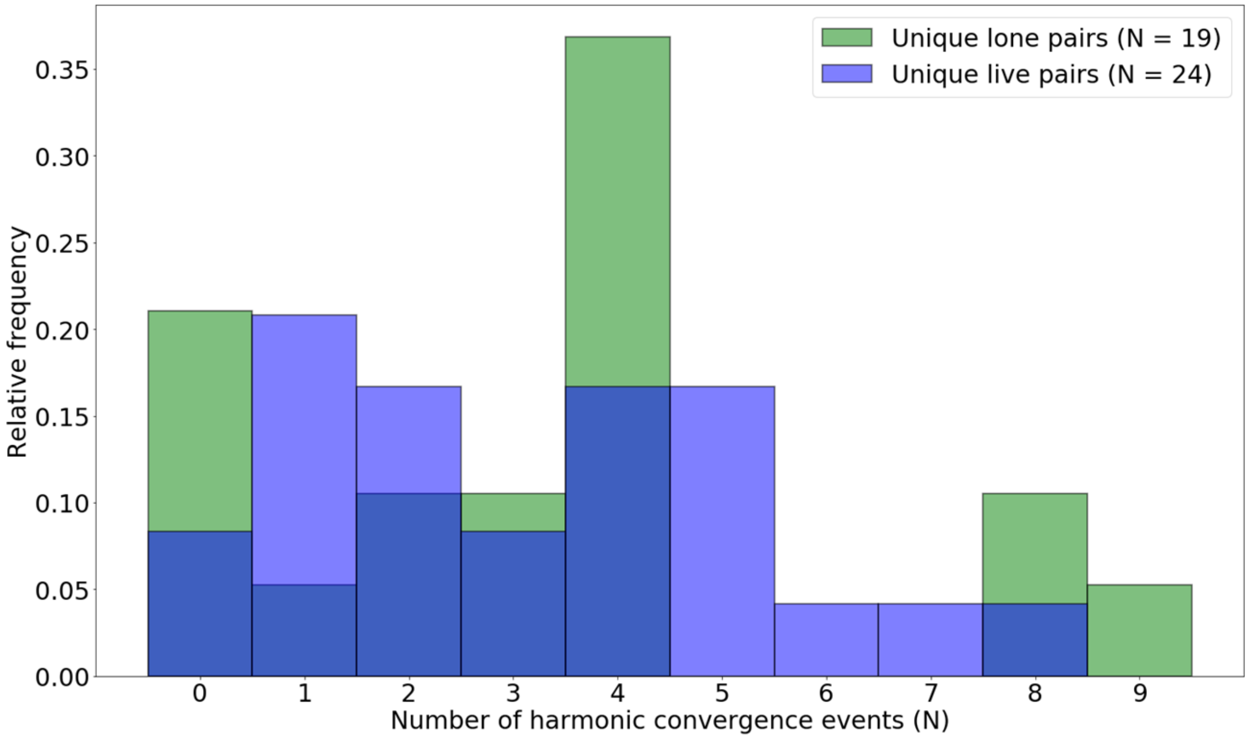

Fig. S15. Superimposed relative frequency distributions of 19 unique *lone* (green), and 24 unique *live* (blue) pairs of mosquitoes are not statistically different. Comparing the distributions of harmonic convergence events between unique lone (=virtual) and unique live (=real) pairs shows no statistically significant differences. Comparison of the datasets used a Mann-Whitney U test, which gave a p-value of 0.98.

1.6 The number of harmonic convergence events as a function of the mean distance from the harmonic convergence ratio

Let us define d_j as the mean of the distances of a pair's ratios from a particular harmonic ratio Hr such that:

$$d_j = mean\left(\left\{Hr - r_{i,j}\right\}_{i=1}^{120}\right)$$

Where Hr can take the values {1, 5/4, 4/3, 3/2, 5/3, 2}. Focusing here on $Hr = 3/2 = 1.5$ (which is the originally proposed and most widely used harmonic convergence ratio (see ref. (*2*)), these distance metrics were calculated for live and lone pairs. In addition, the number of harmonic convergence occurrences at that ratio j, $N_{Hr,j}$, were also counted for all live and lone pairs, chosen such that an occurrence meets criterion (i) of section 1.5. N_{Hr} was then plotted as a function of d for live and lone pairs (Fig. S16).

Criterion (i) for harmonic convergence of section 1.5 states that a harmonic convergence event occurs whenever the male:female frequency ratio $r_{i,j}$ falls within ±1% of any of six ratios {1, 5/4, 4/3, 3/2, 5/3, 2} (though note that here we are only interested in ratio 3/2). Concentrating on this core harmonic convergence criterion, provides a sense of how – for a

given *Hr* - the amount of time a pair spends in a state of convergence is a simple function of the mean distance of the pair's ratios from the respective *Hr* (here: 3/2). Importantly, this perspective has the advantage of being independent of how a harmonic convergence event has been defined by the experimenter in terms of time duration (Fig. S16).

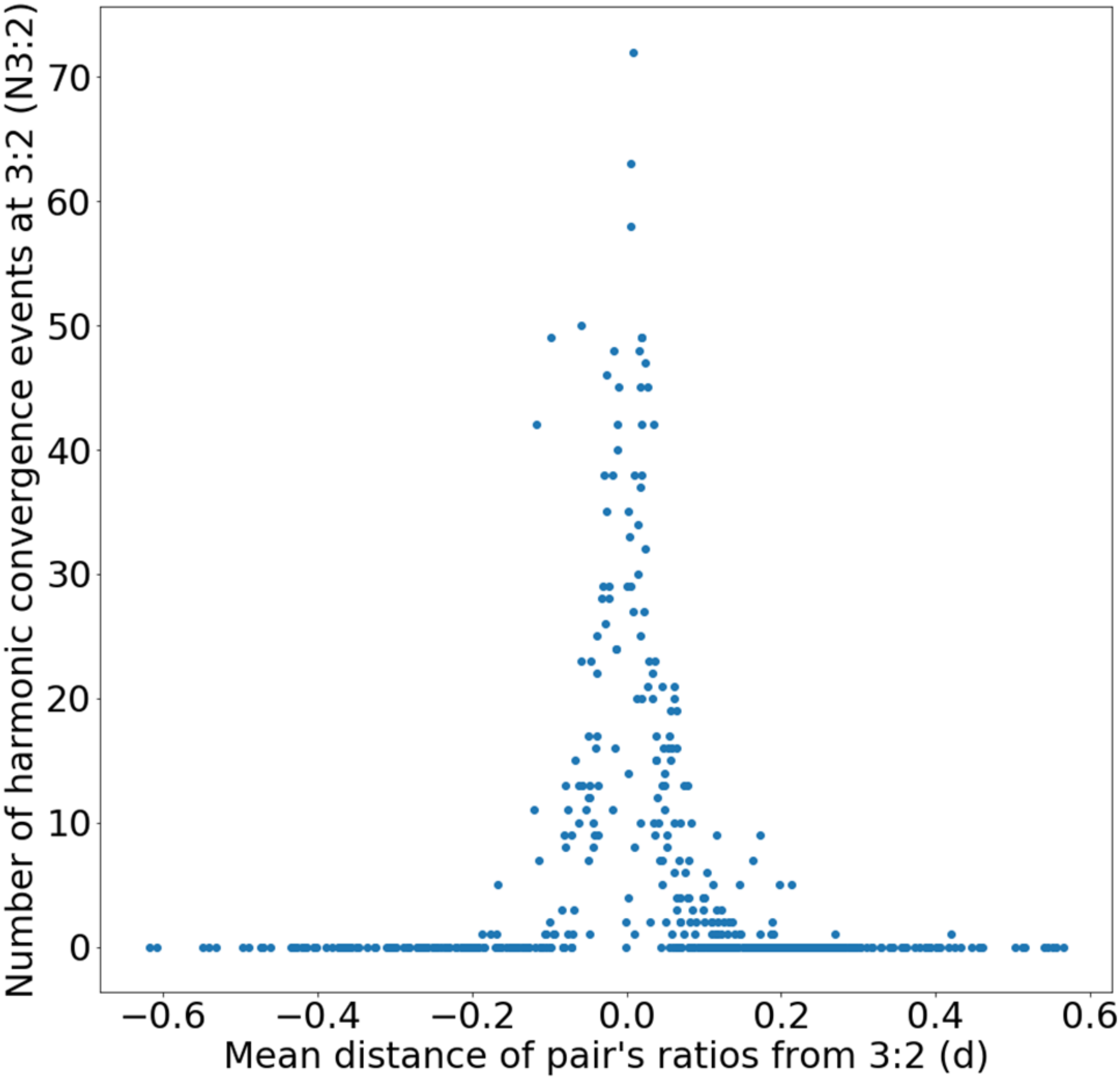

Fig. S16. Number of harmonic convergence events depends critically on a specific pair's mean flight tone ratio. An illustration of how the number of harmonic convergence events at a given ratio (NHr) exhibited by a mosquito pair is a complex function of the mean distance (d) between the pairs' mean frequency ratios and the given harmonic convergence ratio. Note that due to the 1s gap criterion the number of harmonic convergence events drops around zero distances (d=0). All of this introduces a very high sensitivity to noise around the given harmonic convergence ratio. Small random variations of recorded male or female flight

tones (and resulting changes of the flight tone ratios) lead to dramatic increases or decreases in the number of harmonic convergence events, which can bias small sample size data sets.

1.7 Comparison of the distances of pair ratios from the harmonic convergence ratio: Live versus lone pairs

Let us define $d_{a,j}$ as the mean of the absolute distances of a pair's ratios from a harmonic ratio Hr (i.e. the variance of $r_{i,j}$ about Hr) such that:

$$d_{a,j} = mean\left(\{|Hr - r_{i,j}|\}_{i=1}^{120}\right)$$

Where $Hr = 1.5$, as in section 1.6 This distance statistic was computed for each pair of the group of 24 unique live and the group of 19 unique lone pairs of mosquitoes. A comparison of the two groups (Table S1) was conducted using a Mann-Whitney U test (p-value = 0.93).

Table S1. Mean distance of pair ratios from the harmonic convergence ratio 3:2

**Table S1:** Mean distance of pair ratios from the harmonic convergence ratio 3:2	
**Live (*Real*)**	**Lone (*Virtual*)**
0.048	0.264
0.042	0.159
0.021	0.167
0.157	0.401
0.160	0.071
0.130	0.059
0.362	0.163
0.058	0.248
0.192	0.168
0.049	0.092
0.138	0.280
0.118	0.290
0.494	0.264
0.146	0.063
0.561	0.053
0.037	0.134
0.226	0.540
0.230	0.208
0.327	0.070
0.170	
0.170	
0.549	
0.320	
0.487	

1.8 Comparison of flight tone distribution parameters: Live versus lone pair males

The flight tones of each of the 24 males and 24 females that were used in unique live pairs and 27 males and 19 females used in lone pairs were computed as exemplified in section 1.3. Gaussian curves were then fitted on each frequency distribution, extracting the parameters of center, μ, and spread, σ, for each (Table S2). These statistics were subsequently collected for, and compared between, the live and lone groups using Mann-Whitney U tests. Specifically,

four tests were conducted comparing: means (μ) between live males and lone males (p-value = 0.34), standard deviations (σ) between live males and lone males (p-value = 0.74), means between live females and lone females (p-value = 0.08), and standard deviations between live females and lone females (p-value = 0.50).

Table S2. Flight tone distribution of individual mosquitoes (tethered flights, dataset A). Gaussian fit parameters extracted: μ=mean; σ = standard deviation.

**Table S2:** Individual mosquito flight tone distribution Gaussian fit parameter extraction (μ=mean; σ = standard deviation)							
**Live male μ (Hz)**	**Lone male μ (Hz)**	**Live male σ (Hz)**	**Lone male σ (Hz)**	**Live female μ (Hz)**	**Lone female μ (Hz)**	**Live female σ (Hz)**	**Lone female σ (Hz)**
691.5	629.19	23.6	9.9	465.26	509.18	19.59	11.47
600.28	692.52	8.43	8.56	411.82	488.7	6.78	7.65
632.32	806.49	4.24	8.09	423.37	448.76	8.76	7.68
628.13	658.0	3.51	14.21	467.92	489.94	7.96	3.24
593.06	482.69	4.93	3.78	442.83	465.01	7.75	15.63
666.05	641.96	69.98	8.15	448.13	439.35	7.21	4.12
787.84	605.08	13.91	6.06	423.24	452.53	7.11	7.37
696.97	646.27	8.62	3.88	447.41	459.73	6.53	57.58
742.39	671.84	8.89	16.2	438.96	517.53	6.81	23.21
696.91	633.08	23.9	12.21	470.28	463.44	6.94	7.87
709.12	641.96	26.94	8.15	432.92	503.64	6.39	18.1
635.2	745.72	16.06	10.56	459.55	460.15	3.79	25.02
488.97	666.38	8.55	73.42	486.13	462.51	12.21	14.53
728.58	714.65	10.12	11.36	442.62	408.71	4.1	3.1
647.14	566.52	19.89	4.1	689.14	424.51	8.57	11.7
675.69	559.19	7.14	50.23	440.12	433.45	4.33	4.3
686.49	628.76	5.51	14.91	397.96	483.97	9.25	3.91
675.14	861.76	6.61	11.2	390.59	512.57	13.09	22.54
779.56	723.57	12.83	10.85	427.58	505.6	18.05	6.32
869.13	783.56	13.33	11.94	520.85		14.83	
860.44	670.57	9.99	8.98	515.28		7.75	
852.44	618.07	26.28	14.86	416.06		8.57	

652.54	865.51	11.94	13.19	552.99		7.48	
685.71	642.07	9.19	21.56	345.29		7.44	
	836.94		10.64				
	671.13		6.6				
	666.36		8.02				

1.9 Comparison of flight tone diversities: Live versus lone pair males

The *uncertainty* (or it's inverse, the *information*) inherent to a random variable, e.g. the male mosquito's flight tone f_m can be quantified using a measure such as the *Shannon Entropy* of f_m. The Shannon Entropy (also known as Shannon's Diversity Index) (*39*) can be restated, in the context of mosquito flight tones, as a measure of how many frequency channels the mosquito occupies and how often it occupies each of these during its flight. In other words, it is a metric of how diverse a mosquito's landscape of flight tones is. Here the Shannon Entropies of the flight tones of the 24 males and 24 females that were used in unique live pairs and the 27 males and 19 females used in unique lone pairs were computed as follows:

First, the set of each mosquito's flight tones were discretized, and assigned to bins of 5Hz width along the mosquito's flight tone range. Then, the proportion p_{rb} of the number of a male's flight tones n falling in bin b relative to the total number of flight tones is given by:

$$p_{rb} = \frac{n_b}{\sum_b n_b}$$

Then the Shannon entropy H of a mosquito's flight tones is defined us:

$$H \stackrel{\text{def}}{=} -\sum_b p_{rb} \, log_2(p_{rb})$$

The Shannon Entropies of live males' flight tones were compared to those of lone males' flight tones using a t-test (p-values: 0.41, 0.73 for male and female comparisons respectively).

Table S3. Mosquito flight tone diversities (Shannon). Flight tone data from dataset A.

**Calculated flight tone diversities (Shannon)**			
**Live male**	**Lone male**	**Live female**	**Lone female**
3.884	2.839	3.671	2.928
2.686	2.665	2.427	2.642
1.821	2.659	2.412	2.449
1.618	3.375	2.672	1.523

2.022	1.793	2.614	3.45
5.075	2.735	2.473	1.801
3.358	2.325	2.41	2.314
2.791	1.664	2.41	3.229
2.79	3.424	2.466	4.01
3.911	2.618	2.477	2.664
3.944	2.735	2.415	3.198
3.641	2.815	1.735	2.736
2.786	4.149	3.02	3.437
2.85	2.932	1.646	1.494
3.143	1.859	2.813	2.851
2.535	3.023	1.997	1.885
2.157	3.457	2.677	1.711
2.333	3.088	2.738	3.365
3.271	2.957	3.412	2.25
3.151	3.178	3.341	
2.92	2.716	2.608	
4.084	3.303	2.745	
2.945	3.261	2.58	
2.741	3.631	1.83	
	3.026		
	2.376		
	2.651		

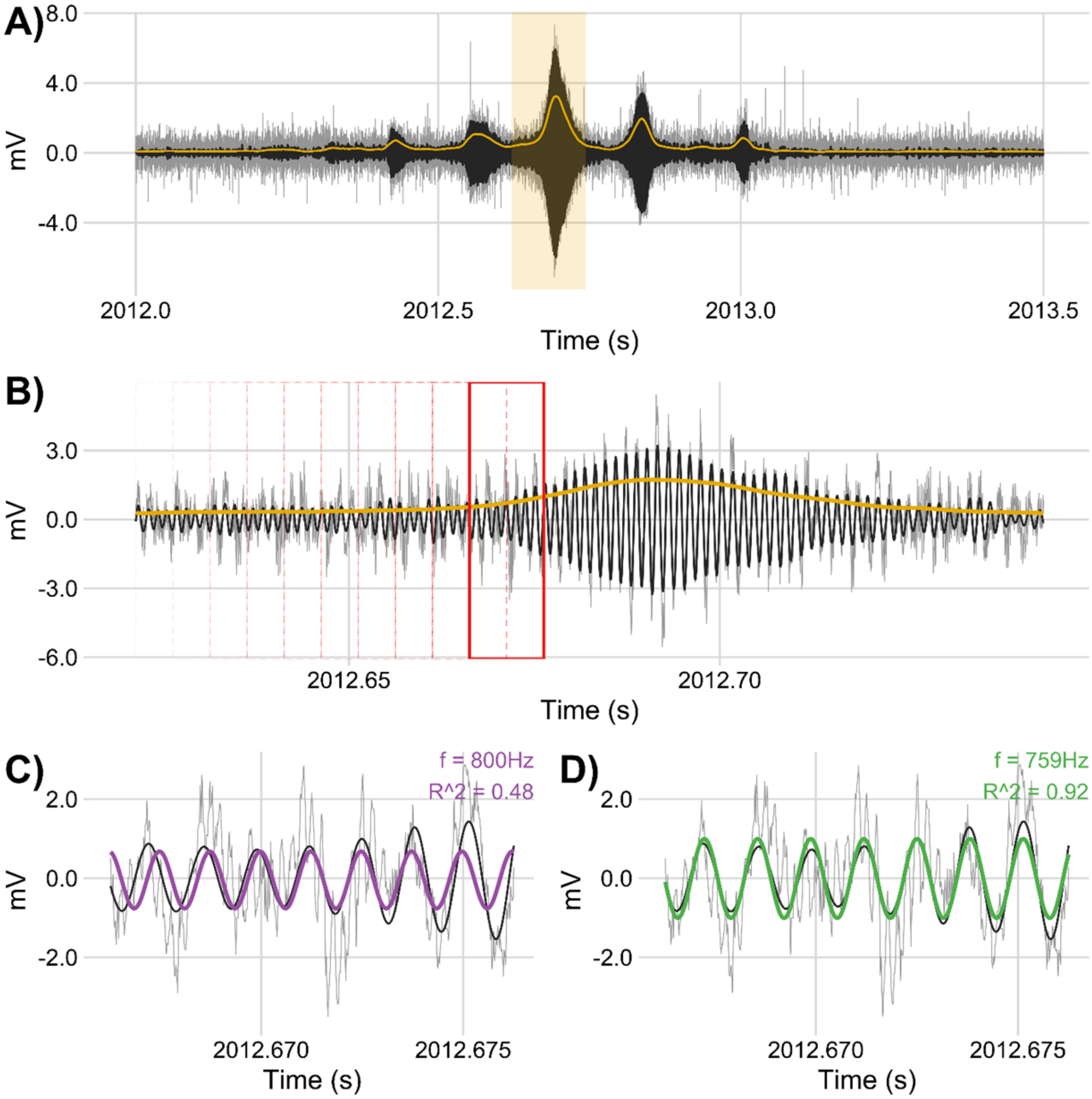

Fig. S17. Flyby event detection pipeline example. A) Raw signal with DC bias correction (light grey line) is filtered through a digital bandpass filter (black line). A moving average envelope is computed (yellow line) to identify flyby events where the envelope is >2*rectified local mean (yellow highlighted region for example). B) Frequency information across the entire flyby event is calculated by fitting data in a sliding window (red rectangles – 10ms window, 50% slide) to a general sinusoidal model (see equation 1 in Methods section).

C) Initial frequency parameter is estimated by fast Fourier transform (purple line) to feed to a optimization algorithm that maximizes the R^2 of the fitted model. D) The frequency of each fitted window, where the R^2 is greater than 0.9 (green line), is then used to calculate the median frequency of each flyby event.

REFERENCES AND NOTES

1. X. Y. Li, H. Kokko, Sex-biased dispersal: A review of the theory. *Biol. Rev.* **94**, 721–736 (2019).

2. W. Takken, C. Costantini, G. Dolo, A. Hassanali, N. Sagnon, E. Osir, Mosquito mating behaviour, in *Bridging Laboratory and Field Research for Genetic Control of Disease Vectors*, B. G. J. Knols, C. Louis, Eds. (Springer, 2006), vol. 11, pp. 183−188.

3. P. I. Howell, B. G. J. Knols, Male mating biology. *Malar. J.* **8**, S8 (2009).

4. J. D. Charlwood, M. D. R. Jones, Mating in the mosquito, *Anopheles gambiae* s.l. *Physiol. Entomol.* **5**, 315–320 (1980).

5. A. Diabaté, A. S. Yaro, A. Dao, M. Diallo, D. L. Huestis, T. Lehmann, Spatial distribution and male mating success of *Anopheles gambiae* swarms. *BMC Evol. Biol.* **11**, 184 (2011).

6. S. B. Poda, C. Nignan, O. Gnankiné, R. K. Dabiré, A. Diabaté, O. Roux, Sex aggregation and species segregation cues in swarming mosquitoes: Role of ground visual markers. *Parasit. Vectors* **12**, 589 (2019).

7. S. P. Sawadogo, A. Niang, E. Bilgo, A. Millogo, H. Maïga, R. K. Dabire, F. Tripet, A. Diabaté, Targeting male mosquito swarms to control malaria vector density. *PLOS ONE* **12**, e0173273 (2017).

8. E. W. Kaindoa, *H. S. Ngowo, A. J. Limwagu, M. Tchouakui*, E. Hape, S. Abbasi, J. Kihonda, A. S. Mmbando, R. M. Njalambaha, G. Mkandawile, H. Bwanary, M. Coetzee, F. O. Okumu, Swarms of the malaria vector *Anopheles funestus* in Tanzania. *Malar. J.* **18**, 29 (2019).

9. J. D. Charlwood, R. Thompson, H. Madsen, Observations on the swarming and mating behaviour of *Anopheles funestus* from southern Mozambique. *Malar. J.* **2**, 2 (2003).

10. C. Montell, L. J. Zwiebel, in *Progress in Mosquito Research*, A. S. Raikhel, Ed. (Academic Press Ltd.–Elsevier Science Ltd., 2016), vol. 51, pp. 293–328.

11. C. Johnston, Auditory apparatus of the *Culex* mosquito. *J. Cell Sci.* **3**, 97–102 (1855).

12. M. C. Göpfert, D. Robert, Active auditory mechanics in mosquitoes. *Proc. R. Soc. B Biol. Sci.* **268**, 333–339 (2001).

13. J. T. Albert, A. S. Kozlov, Comparative aspects of hearing in vertebrates and insects with antennal ears. *Curr. Biol.* **26**, R1050–R1061 (2016).

14. M. C. Göpfert, H. Briegel, D. Robert, Mosquito hearing: Sound-induced antennal vibrations in male and female *Aedes aegypti*. *J. Exp. Biol.* **202**, 2727–2738 (1999).

15. M. C. Göpfert, D. Robert, Nanometre-range acoustic sensitivity in male and female mosquitoes. *Proc. Biol. Sci.* **267**, 453–457 (2000).

16. M. P. Su, M. Andrés, N. Boyd-Gibbins, J. Somers, J. T. Albert, Sex and species specific hearing mechanisms in mosquito flagellar ears. *Nat. Commun.* **9**, 3911 (2018).

17. G. Gibson, B. Warren, I. J. Russell, Humming in tune: Sex and species recognition by mosquitoes on the wing. *J. Assoc. Res. Otolaryngol.* **11**, 527–540 (2010).

18. F. Julicher, D. Andor, T. Duke, Physical basis of two-tone interference in hearing. *Proc. Natl. Acad. Sci. U.S.A.* **98**, 9080–9085 (2001).

19. B. Warren, G. Gibson, I. J. Russell, Sex recognition through midflight mating duets in *Culex* mosquitoes is mediated by acoustic distortion. *Curr. Biol.* **19**, 485–491 (2009).

20. P. M. V. Simões, R. Ingham, G. Gibson, I. J. Russell, Masking of an auditory behaviour reveals how male mosquitoes use distortion to detect females. *Proc. R. Soc. B* **285**, 20171862 (2018).

21. L. J. Cator, B. J. Arthur, L. C. Harrington, R. R. Hoy, Harmonic convergence in the love songs of the dengue vector mosquito. *Science* **323**, 1077–1079 (2009).

22. A. Aldersley, A. Champneys, M. Homer, D. Robert, Quantitative analysis of harmonic convergence in mosquito auditory interactions. *J. R. Soc. Interface* **13**, 20151007 (2016).

23. G. Wang, J. Vega-Rodríguez, A. Diabate, J. Liu, C. Cui, C. Nignan, L. Dong, F. Li, C. O. Ouedrago, A. M. Bandaogo, P. S. Sawadogo, H. Maiga, T. L. Alves e Silva, T. V. Pascini, S. Wang, M. Jacobs-

Lorena, Clock genes and environmental cues coordinate *Anopheles* pheromone synthesis, swarming, and mating. *Science* **371**, 411–415 (2021).

24. S. M. Villarreal, O. Winokur, L. Harrington, The impact of temperature and body size on fundamental flight tone variation in the mosquito vector *Aedes aegypti* (Diptera: Culicidae): Implications for acoustic lures. *J. Med. Entomol.* **54**, 1116–1121 (2017).

25. T. Nakata, N. Phillips, P. Simões, I. J. Russell, J. A. Cheney, S. M. Walker, R. J. Bomphrey, Aerodynamic imaging by mosquitoes inspires a surface detector for autonomous flying vehicles. *Science* **368**, 634−637 (2020).

26. P. M. V. Simões, R. A. Ingham, G. Gibson, I. J. Russell, A role for acoustic distortion in novel rapid frequency modulation behaviour in free-flying male mosquitoes. *J. Exp. Biol.* **219**, 2039–2047 (2016).

27. D. N. Lapshin, D. D. Vorontsov, Frequency tuning of swarming male mosquitoes (*Aedes communis*, Culicidae) and its neural mechanisms. *J. Insect Physiol.* **132**, 104233 (2021).

28. C. Pennetier, B. Warren, K. R. Dabiré, I. J. Russell, G. Gibson, "Singing on the Wing" as a mechanism for species recognition in the malarial mosquito *Anopheles gambiae*. *Curr. Biol.* **20**, 131–136 (2010).

29. M. P. Su, M. Georgiades, J. Bagi, K. Kyrou, A. Crisanti, J. T. Albert, Assessing the acoustic behaviour of *Anopheles gambiae* (s.l.) dsxF mutants: Implications for vector control. *Parasit. Vectors* **13**, 507 (2020).

30. A. N. Clements, *The Biology of Mosquitoes. Vol. 2: Sensory Reception and Behaviour* (CABI Publishing, 1999).

31. L. O. Araripe, J. R. A. Bezerra, G. B. da Silva Rivas, R. V. Bruno, Locomotor activity in males of *Aedes aegypti* can shift in response to females' presence. *Parasit. Vectors* **11**, 254 (2018).

32. L. J. Cator, L. C. Harrington, The harmonic convergence of fathers predicts the mating success of sons in *Aedes aegypti*. *Anim. Behav.* **82**, 627–633 (2011).

33. A. Qureshi, A. Aldersley, B. Hollis, A. Ponlawat, L. J. Cator, Male competition and the evolution of mating and life-history traits in experimental populations of *Aedes aegypti*. *Proc. R. Soc. B* **286**, 20190591 (2019).

34. Z. W. Dou, A. Madan, J. S. Carlson, J. Chung, T. Spoleti, G. Dimopoulos, A. Cammarato, R. Mittal, Acoustotactic response of mosquitoes in untethered flight to incidental sound. *Sci. Rep.* **11**, 1884 (2021).

35. R. J. Bomphrey, T. Nakata, N. Phillips, S. M. Walker, Smart wing rotation and trailing-edge vortices enable high frequency mosquito flight. *Nature* **544**, 92–95 (2017).

36. Q. Geissmann, L. Garcia Rodriguez, E. J. Beckwith, G. F. Gilestro, Rethomics: An R framework to analyse high-throughput behavioural data. *PLOS ONE* **14**, e0209331 (2019).

37. G. Gibson, I. Russell, Flying in tune: Sexual recognition in mosquitoes. *Curr. Biol.* **16**, 1311–1316 (2006).

38. A. Aldersley, A. Champneys, M. Homer, D. Robert, Time-frequency composition of mosquito flight tones obtained using Hilbert spectral analysis. *J. Acoust. Soc. Am.* **136**, 1982–1989 (2014).

39. C. E. Shannon, W. Weaver, *The Mathematical Theory of Communication* (University of Illinois Press, 1949).
